# Fecal microbiota transplantation for the improvement of metabolism in obesity: The FMT-TRIM double-blind placebo-controlled pilot trial

**DOI:** 10.1371/journal.pmed.1003051

**Published:** 2020-03-09

**Authors:** Elaine W. Yu, Liu Gao, Petr Stastka, Michael C. Cheney, Jasmin Mahabamunuge, Mariam Torres Soto, Christopher B. Ford, Jessica A. Bryant, Matthew R. Henn, Elizabeth L. Hohmann

**Affiliations:** 1 Endocrine Unit, Division of Endocrinology and Metabolism, Massachusetts General Hospital, Boston, Massachusetts, United States of America; 2 Harvard Medical School, Boston, Massachusetts, United States of America; 3 Division of Infectious Diseases, Massachusetts General Hospital, Boston, Massachusetts, United States of America; 4 Seres Therapeutics, Cambridge, Massachusetts, United States of America; Harvard Medical School, UNITED STATES

## Abstract

**Background:**

There is intense interest about whether modulating gut microbiota can impact systemic metabolism. We investigated the safety of weekly oral fecal microbiota transplantation (FMT) capsules from healthy lean donors and their ability to alter gut microbiota and improve metabolic outcomes in patients with obesity.

**Methods and findings:**

FMT-TRIM was a 12-week double-blind randomized placebo-controlled pilot trial of oral FMT capsules performed at a single US academic medical center. Between August 2016 and April 2018, we randomized 24 adults with obesity and mild–moderate insulin resistance (homeostatic model assessment of insulin resistance [HOMA-IR] between 2.0 and 8.0) to weekly healthy lean donor FMT versus placebo capsules for 6 weeks. The primary outcome, assessed by intention to treat, was change in insulin sensitivity between 0 and 6 weeks as measured by hyperinsulinemic euglycemic clamps. Additional metabolic parameters were evaluated at 0, 6, and 12 weeks, including HbA1c, body weight, body composition by dual-energy X-ray absorptiometry, and resting energy expenditure by indirect calorimetry. Fecal samples were serially collected and evaluated via 16S V4 rRNA sequencing. Our study population was 71% female, with an average baseline BMI of 38.8 ± 6.7 kg/m^2^ and 41.3 ± 5.1 kg/m^2^ in the FMT and placebo groups, respectively. There were no statistically significant improvements in insulin sensitivity in the FMT group compared to the placebo group (+5% ± 12% in FMT group versus −3% ± 32% in placebo group, mean difference 9%, 95% CI −5% to 28%, *p =* 0.16). There were no statistically significant differences between groups for most of the other secondary metabolic outcomes, including HOMA-IR (mean difference 0.2, 95% CI −0.9 to 0.9, *p =* 0.96) and body composition (lean mass mean difference −0.1 kg, 95% CI −1.9 to 1.6 kg, *p =* 0.87; fat mass mean difference 1.2 kg, 95% CI −0.6 to 3.0 kg, *p =* 0.18), over the 12-week study. We observed variable engraftment of donor bacterial groups among FMT recipients, which persisted throughout the 12-week study. There were no significant differences in adverse events (AEs) (10 versus 5, *p =* 0.09), and no serious AEs related to FMT. Limitations of this pilot study are the small sample size, inclusion of participants with relatively mild insulin resistance, and lack of concurrent dietary intervention.

**Conclusions:**

Weekly administration of FMT capsules in adults with obesity results in gut microbiota engraftment in most recipients for at least 12 weeks. Despite engraftment, we did not observe clinically significant metabolic effects during the study.

**Trial registration:**

ClinicalTrials.gov NCT02530385.

## Abstract 2

Elain W Yu et al investigate the potential of fecal microbiota transplantation to improve metabolism in obese individuals.

## Author summary

**Why was this study done?:**

- Animal studies show that body weight and glycemic regulation can be markedly altered by manipulation of the intestinal microbiota.
- Two prior studies in men with obesity and metabolic syndrome showed that a single nasoduodenal fecal microbiota transplantation (FMT) from lean donors led to small and transient improvements in glycemic outcomes.
- We hypothesized that repeated dosing with oral FMT capsules from lean donors could lead to lasting improvements in metabolic outcomes.

**What did the researchers do and find?:**

- We randomized adults with obesity who were at high risk for development of type 2 diabetes to receive either weekly oral FMT capsules from healthy lean donors or placebo capsules for 6 weeks.
- Oral FMT was safe and tolerable, and we observed durable microbial shifts in most participants receiving FMT.
- We found no significant differences between groups in most glycemic outcomes, weight, or body composition over a 12-week period. There was a minor improvement in HbA1c after FMT as compared to placebo.
- Exploratory analyses suggest possible improvement in metabolism after FMT among study participants with low baseline microbiome diversity, similar to what was observed in a prior study.

**What do these findings mean?:**

- Our results suggest that intestinal microbial manipulation by FMT capsules does not meaningfully alter human metabolism and weight in adults with obesity.
- Regional differences between study populations, differences in route of FMT administration, and differences in engrafting microbial species all might explain the overall negative findings and discordance with prior trials.
- Future studies should evaluate pre-selection of donors and recipients, and consider microbiome and lifestyle modifications concurrently.

## Introduction

There has been much excitement about the potential role of the gut microbiome in influencing systemic metabolism and the development of diabetes and other cardiometabolic disorders [1,2]. The relationship between obesity, diet, metabolic diseases, and the microbiome is complex, and despite intense interest in this topic, there are few clinical studies to establish causality. The most intriguing data are derived from preclinical mouse models, which have demonstrated that genetic-, diet-, and medication-induced obesity result in microbiome shifts that confer susceptibility to obesity and negative metabolic outcomes when transferred to a new host [3–11]. Remarkably, weight gain has been shown to occur without mice switching to an obesity-inducing diet, suggesting an outsized role of the gut microbiome in dictating body weight. However, the germ-free and conventional animal models used in these studies do not directly replicate human diet, microbiome, or gastrointestinal physiology [12], and therefore these provocative results require clinical validation in human studies.

In humans, the potential relationships between obesity, metabolic disease, and the microbiome are less clear. Antibiotic exposure early in life increases later risk for obesity, presumably via undesirable alterations in the gut microbiome during childhood development [13,14]. Initial small cohort studies suggested that adults with obesity [15–17] and those with type 2 diabetes [18,19] have a different gut microbiome signature than lean controls, with decreased bacterial and/or genetic diversity. Larger cross-sectional cohorts of >1,000 patients showed mixed results, with some finding no consistent diversity or compositional differences between lean and obese adults [20] and others noting small but significant associations between microbiome and body mass index, metabolism, and body composition [21,22].

The most provocative clinical evidence supporting the metabolic potential of microbiota alterations is derived from 2 randomized clinical trials from the Netherlands. In a pilot study with 18 participants (*n =* 9 FMT recipients, *n =* 9 controls), and a subsequent larger follow-up study with 38 participants (*n =* 26 FMT recipients, *n =* 12 controls), one research group has observed that fecal microbiota transplantation (FMT) from lean donors can transiently improve peripheral insulin sensitivity in men with obesity and metabolic syndrome [23,24]. Nevertheless, these studies found reversion of the gut microbiome and insulin resistance in the FMT recipients back to baseline within 12–18 weeks after FMT administration, indicating a short-lived effect.

Prior clinical studies of FMT have typically relied on direct administration of fresh stool suspensions via upper or lower endoscopic procedures (e.g., esophagogastroduodenoscopy, colonoscopy), often with preceding gastrointestinal lavage and in the setting of pretreatment antibiotics [25]. While these procedures are effective delivery systems, their moderate invasiveness limits considerations of repeated FMT administrations. We have pioneered a novel encapsulation technique to safely deliver oral encapsulated frozen FMT inocula in a clinical setting [26]. FMT capsules have proven to be as efficacious as endoscopically delivered FMT for recurrent *Clostridium difficile* colitis, and with better patient acceptability ratings [27]. Encapsulated FMT is now offered as standard care for recurrent *C*. *difficile* at our institution, and over 400 patients (including 202 formally reported [28]) have been treated without serious related adverse events (AEs).

Given the excellent safety and tolerability profile of FMT capsules, we sought to investigate whether repeated FMT administration could be a viable treatment strategy for durably modifying the gut microbiome and improving human metabolism. We therefore conducted a pilot double-blind randomized placebo-controlled trial that involved weekly administration of oral FMT capsules derived from healthy lean donors delivered to adults with obesity and mild–moderate insulin resistance. We hypothesized that weekly oral FMT would (1) safely and sustainably alter the microbiome among recipients with obesity and (2) improve metabolic endpoints, including insulin sensitivity as assessed by hyperinsulinemic euglycemic clamps.

## Methods

### Study design

FMT-TRIM was a 12-week double-blind randomized placebo-controlled pilot trial of encapsulated frozen FMT from healthy lean donors to adults with obesity and insulin resistance that was conducted at a single US academic medical center. Between August 25, 2016, and April 4, 2018, we recruited 24 study participants aged 25–60 years with BMI ≥ 30 kg/m^2^ and mild to moderate insulin resistance, defined as having a homeostatic model assessment of insulin resistance (HOMA-IR) between 2.0 and 8.0. Exclusion criteria were antibiotic use in the prior 6 months, established diabetes, use of medications known to affect body weight or insulin sensitivity in the past 3 months, gastrointestinal or malabsorptive disorders, immunosuppression, and significant liver or renal disease. Information about donor screening, FMT and placebo capsule preparation, and other protocol details can be found in S1 Methods.

Study participants with obesity were randomized 1:1 to receive frozen FMT capsules or frozen placebo capsules. Randomization was performed by computer-generated random sequence in blocks of 4. Participants were not given any preparatory bowel cleansing but were instructed to fast for 4 hours prior to and 1 hour following capsule administrations. A bowel preparation was intentionally not included, for participant convenience and to assess whether the microbiome could be durably shifted without this procedure. Weekly oral capsule administrations were performed by study staff in a monitored clinical setting. At baseline (i.e., week 0), participants were administered 15 capsules on each of 2 consecutive days, followed by 15 capsules once a week for the next 5 weeks. Although there were multiple donors for the study, each FMT participant only received capsules from a single donor. At each capsule administration and study visit, study staff performed an interviewer-administered targeted assessment to assess potential AEs since the prior visit, which were graded in accordance with Common Terminology Criteria for Adverse Events (CTCAE) v4.0. Specific AEs assessed for included fever, diarrhea, nausea/vomiting, fatigue/malaise, headache, and distention/bloating/abdominal pain or discomfort. Study participants, investigators, and outcome assessors were masked to group assignment throughout the study. Study participants were asked to maintain a stable dietary and physical activity pattern throughout the 12-week study.

The trial was approved by the Partners Human Research Committee (protocol 2015P001632), and all study participants and donors provided written informed consent. The trial is registered on ClinicalTrials.gov (NCT02530385).

### Metabolic outcomes

The primary outcome of insulin sensitivity was measured by insulin-stimulated glucose uptake (M value) during hyperinsulinemic euglycemic clamps at 0 and 6 weeks (details available in S1 Methods). Secondary metabolic measurements were performed at 0, 6, and 12 weeks, unless otherwise noted. Height and weight were measured in triplicate using a wall-mounted stadiometer (Harpenden, Seritex) and digital scale (Tanita BWB-800, Tanita Corporation of America), respectively. Dietary intake data were collected and analyzed using Nutrition Data System for Research software (Nutrition Coordinating Center, University of Minnesota). Body composition was measured by dual-energy X-ray absorptiometry (DXA) whole body scans (Hologic Discovery A), which provided subtotal body (i.e., total body excluding head) measurements of fat mass (kg), lean mass (kg), percent fat (%), and visceral adipose tissue (cm^3^). Resting energy expenditure measurement was performed at 0 and 6 weeks and estimated via indirect calorimetry using the VMAX 29 Encore Metabolic Cart (Vyaire Medical). Fasting blood was collected at 0, 6, and 12 weeks and stored at −80°C. Fasting glucose, hemoglobin A1c (HbA1c), lipids, and C-reactive protein were measured by standard clinical assays (Labcorp). Serum insulin was assessed by radioimmunoassay (Human Insulin-Specific RIA Kit, Millipore Corporation; inter-assay coefficient of variation 5.2%).

### Microbiome assessments

Stool samples were collected from study participants at 0, 1, 3, 5, 6, and 12 weeks. A detailed description of microbiome sequencing, data processing, and donor stool sampling is provided in S1 Methods. Briefly, participants produced samples within 12 hours prior to each study visit and stored samples in an insulated transport container with frozen gel packs until delivered to the study staff. Donor preparations and study participant samples were characterized by 16S V4 amplicon sequencing. The set of unique 16S V4 DNA sequences, referred to as amplicon sequence variants (ASVs), was then inferred using the Dada2 algorithm. The Silva database was used for taxonomic assignments [29].

Additionally, we sequenced a subset of donor 1 FMT recipient and placebo samples using shotgun metagenomics, for finer taxonomic resolution. Baseline and week 1, 6, and 12 samples from the 3 donor 1 FMT recipients, 3 placebo participants, and donor 1 preparations (preps) were sequenced with shotgun metagenomics sequencing (whole metagenome sequencing [WMS]) using a Nextera DNA Flex Library Prep Kit on an Illumina HiSeq platform. We used the Metaphlan2 package to infer species-level taxonomic composition of all 24 WMS samples and Strainphlan (version 1.2.0 with parameter:–relaxed3) to infer strains and phylogenetic distances between strains of the same species.

### Statistical analysis

Assuming a standard deviation of 30%–45% for insulin sensitivity change and a 2-sided alpha of 0.05, this pilot trial (*n =* 12 per group) had 80% power to detect a 40%–60% difference in insulin sensitivity between groups, allowing for a possible 15% dropout rate. Baseline characteristics were compared between groups using independent *t* test, Wilcoxon rank sum test, Fisher’s exact test, or chi-squared test, as appropriate. Data were analyzed according to intention-to-treat principles. Results are reported as mean ± standard deviation (SD) for normal data and median [Q1, Q3] for non-normal data. Our primary outcome was comparison of the percentage change in insulin sensitivity (M value) from 0 to 6 weeks in the FMT and placebo groups. Due to the presence of outliers for this outcome, we analyzed data using a Wilcoxon rank sum test. Prespecified secondary outcomes included changes between baseline and 12 weeks for the following measures: HOMA-IR, body weight, lean mass as assessed by DXA, and fat mass as assessed by DXA. All other outcomes reported were exploratory. For outcomes measured at more than 2 timepoints (e.g., 0, 6, and 12 weeks), a longitudinal general linear mixed effects model (SAS PROC MIXED) with a compound symmetry covariance structure was used to compare change in secondary metabolic outcomes between the FMT and placebo groups over the 12-week study. The participant-specific intercept was considered a random effect, and time, group, and time × group interaction were considered fixed effects. We also performed a sensitivity analysis of the primary outcome (insulin sensitivity) using the linear mixed effects model with adjustment for the baseline value. Finally, for 1 study participant in the placebo group, the baseline insulin clamp was disrupted by a fire alarm; although data were deemed valid to keep in the main analysis, an additional sensitivity analysis excluding this outlier was also performed. Analyses of metabolic endpoints were performed using SAS 9.4 software (SAS Institute).

Microbiome alpha and beta diversity were assessed by applying the Shannon diversity index and UniFrac dissimilarity metrics [30] to 16S V4 DNA sequencing data as detailed in S1 Methods. For engraftment analysis, each ASV was considered in the context of participant–donor pairings. If an ASV was identified in any prep from the participant’s paired donor material and any of the participant’s post-dosing samples (week 1–12), but not in the participant’s baseline sample, the donor-specific ASV was considered an “engrafting” ASV. If an ASV was identified in a baseline participant sample, but not in any preps from the paired donor, it was considered a “participant-specific” ASV. If an ASV was observed in both the participant baseline sample and any paired donor prep, it was considered “common to participant and donor.” ASVs that were only observed in participants following dosing were labeled “newly detected.” Heatmaps displaying the dynamics of engrafting ASVs were generated using the ComplexHeatmap R package [31]. To display ASV abundances in heatmaps, ASV counts for each prep were subsampled to the same sequencing depth, a pseudocount of 1 was applied, values were transformed to relative abundances, and finally values were log10 transformed. To estimate enterotype status, we calculated the ratio of Prevotellaceae to Bacteroidaceae abundance. Family-level resolution instead of the more traditional genus-level resolution was used because the Silva taxonomy contains 15 *Prevotella* subgroups instead of a single genus.

The significance of correlations between changes in microbiome community composition and changes in metabolic measurements was quantified using Mantel tests with Spearman correlation coefficients [32]. Percentage change in ASV richness and diversity from baseline to the end of the dosing period between the FMT and placebo groups was evaluated using Wilcoxon rank sum tests. In exploratory post hoc analyses, we examined whether baseline microbial diversity influenced response to treatment, as previously suggested [24]. After excluding participants with baseline microbiome diversity above the baseline median (Shannon diversity index of 3.1), we replicated the longitudinal general linear mixed effects model (FMT *n = 5*, placebo *n* = 7) comparing clinical changes between the FMT and placebo groups throughout the 12-week study.

## Results

**Fig 1:**
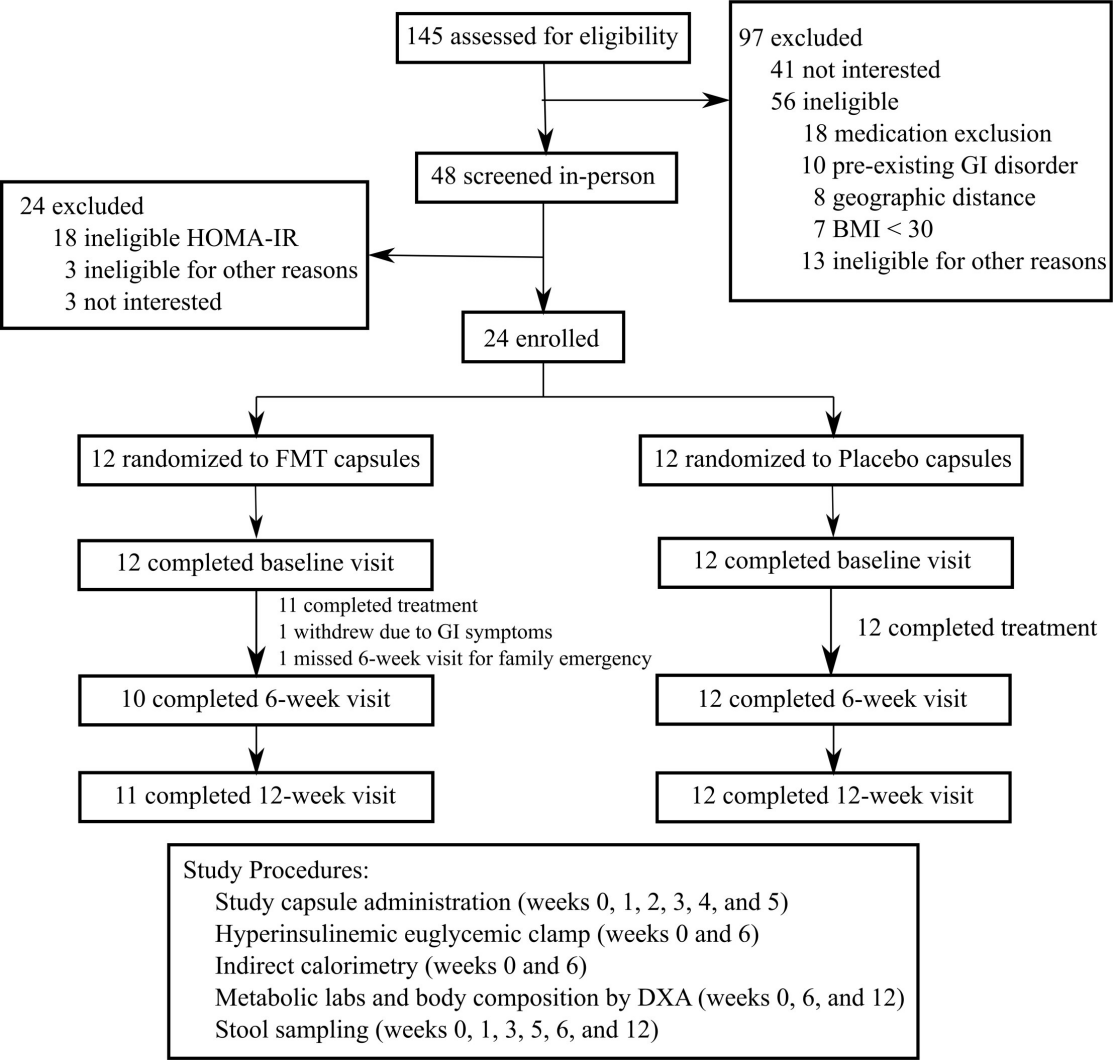
Study flow diagram. DXA, dual-energy X-ray absorptiometry; FMT, fecal microbiota transplantation; GI, gastrointestinal; HOMA-IR, homeostatic model assessment of insulin resistance.

Between August 2016 and April 2018, we screened 145 individuals to recruit 24 adults with obesity and mild–moderate insulin resistance to participate in this randomized controlled trial (Fig 1). At baseline, the FMT and placebo groups were well balanced in terms of age, sex, weight, and bionutritional measures (Table 1). Our study population was predominantly female, with an average BMI of 38.8 ± 6.7 kg/m^2^ and 41.3 ± 5.1 kg/m^2^ in the FMT and placebo groups, respectively. Of the 24 randomized participants, 23 (96%) completed the 12-week study, including all weekly supervised capsule administrations. Within the FMT group, 1 participant dropped out after week 2 due to gastrointestinal symptoms and did not attend subsequent study visits, and 1 participant missed the 6-week visit due to a family emergency but attended the 12-week visit. Four metabolically healthy lean donors (3 women, 1 man; BMI range 19.5–21.8 kg/m^2^) provided material for the FMT capsules that were delivered to 12 FMT recipients, with a range of 1 to 5 recipients per donor. The baseline characteristics of the donors are shown in Table A in S1 Data.

**Table 1:** Baseline characteristics of randomized participants.

Characteristic	Placebo group	FMT group
*N*	12	12
Age (y)	38.5 ± 8.8	42.5 ± 8.4
Female, *N* (%)	9 (75%)	8 (67%)
Race, *N* (%)		
Black non-Hispanic	1 (8%)	1 (8%)
White non-Hispanic	10 (83%)	9 (75%)
White Hispanic	1 (8%)	2 (16%)
BMI (kg/m^2^)	41.3 ± 5.1	38.8 ± 6.7
Weight (kg)	111 ± 20	110 ± 26
Height (cm)	164 ± 9	168 ± 10
Lean mass (kg)	58 ± 12	60 ± 15
Fat mass (kg)	53 ± 10	49 ± 13
VAT volume (cm^3^)	998 ± 319	1,048 ± 368
Fasting glucose (mmol/l)	4.8 ± 0.4	5.0 ± 0.7
Fasting insulin (pmol/l)	116.7 ± 63.9	109.7 ± 38.9
HOMA-IR	3.5 ± 1.9	3.5 ± 1.4
M value (mg/kg/min)	7.4 [5.3, 9.6]	6.4 [5.3, 7.0]
HbA1c (%)	5.5 ± 0.3	5.6 ± 0.2
Total cholesterol (mmol/l)	5.1 ± 0.6	5.5 ± 0.6
LDL cholesterol (mmol/l)	3.2 ± 0.6	3.3 ± 0.8
HDL cholesterol (mmol/l)	1.2 ± 0.3	1.3 ± 0.4
Triglycerides (mmol/l)	1.3 [1.1, 1.8]	1.7 [1.1, 2.2]
CRP (mg/l)	3.5 [2.3, 7.3]	2.9 [1.7, 5.6]
REE (kcal/day)	1,503 ± 218	1,588 ± 305
Caloric intake (kcal/day)	1,939 ± 463	2,121 ± 729

Data are *n* (%), mean ± SD, or median [Q1, Q3].

BMI, body mass index; CRP, C-reactive protein; FMT, fecal microbiota transplantation; HbA1c, hemoglobin A1c; HDL, high-density lipoprotein; HOMA-IR, homeostatic model assessment of insulin resistance; LDL, low-density lipoprotein; REE, resting energy expenditure; VAT, visceral adipose tissue.

**Fig 2:**
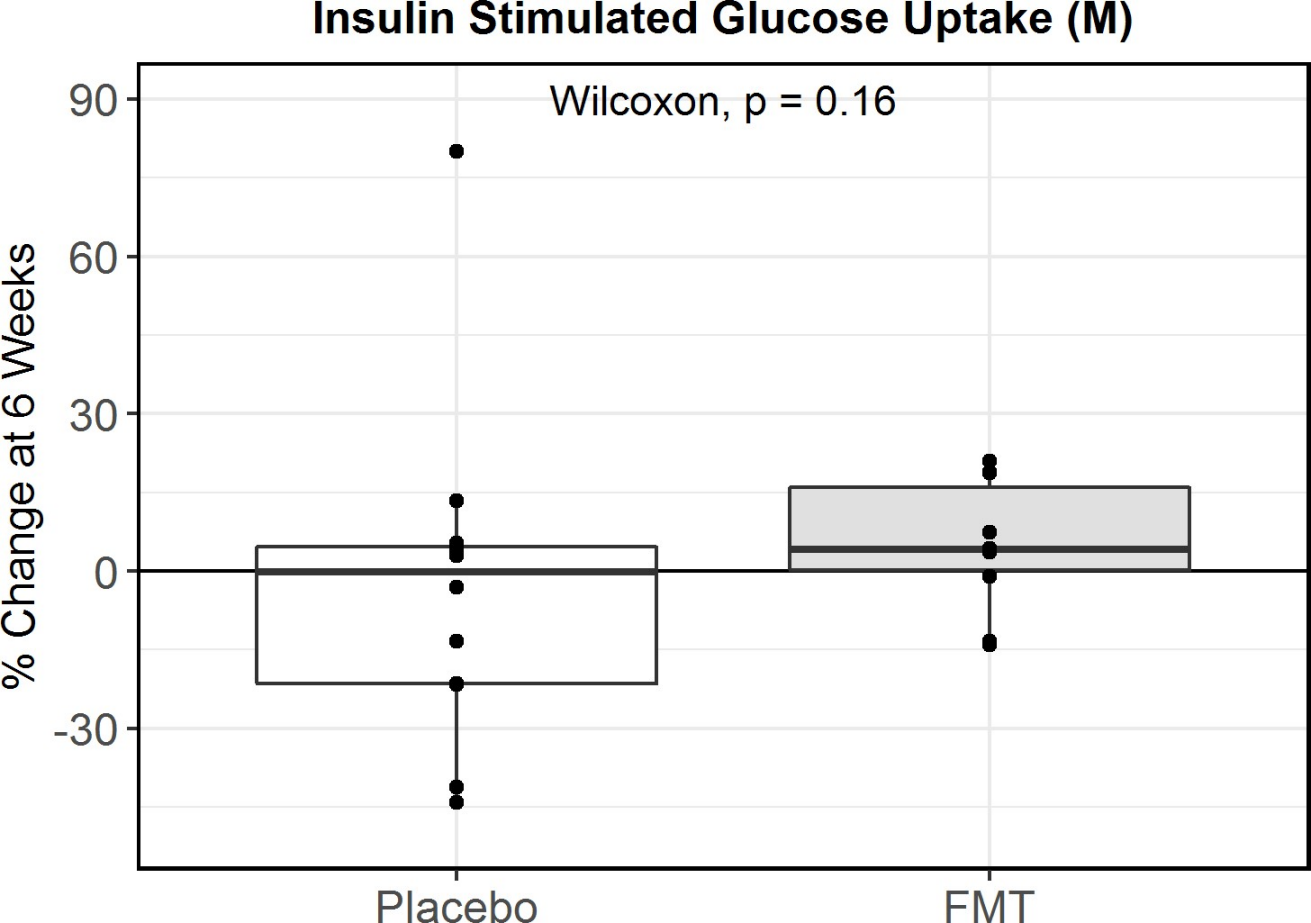
Boxplot of percentage change in insulin sensitivity in fecal microbiota transplantation (FMT) and placebo groups from baseline to 6 weeks. Insulin-stimulated glucose uptake (M value) was assessed by hyperinsulinemic euglycemic clamp as a measurement of insulin sensitivity.

After 6 weeks of treatment with study capsules, there were nonsignificant improvements in insulin sensitivity in the FMT group as compared to the placebo group (percentage change in insulin-stimulated glucose uptake: +5% ± 12% FMT versus −3% ± 32% placebo; mean difference 9%, 95% CI −5% to 28%; *p =* 0.16; Fig 2). Results were similar when insulin-stimulated glucose uptake was corrected for steady-state insulin level (Fig A in S1 Data, *p =* 0.14). Furthermore, sensitivity analysis using a linear mixed model with adjustment for baseline insulin sensitivity yielded a similarly nonsignificant difference between groups (*p =* 0.46). Exclusion of the outlier in the placebo group led to a suggestion of improvement in insulin sensitivity in the FMT group as compared to the placebo group that nevertheless was not statistically significant (mean difference 14%, 95% CI −1% to 30%, *p =* 0.06).

There were no differences between the FMT and placebo groups in change in HOMA-IR, fat mass, fasting lipids, or resting energy expenditure over the 12-week study (Table 2). Body weight was similarly unchanged throughout the study. There was a statistically significant but clinically minor greater reduction in HbA1c at 12 weeks (mean difference −0.1%, 95% CI −0.3 to −0.01%, *p =* 0.04) and increase in C-reactive protein at 6 weeks (mean difference 1.8 mg/l, 95% CI 0.3 to 3.3, *p =* 0.02) in the FMT group as compared to the placebo group (Table 2). There were subtle changes in caloric intake throughout the study, but these were not statistically different between groups (Table 2). In exploratory post hoc analyses, change in insulin-stimulated glucose uptake did not appear to differ by donor (*p =* 0.88) or by baseline demographics (sex, *p =* 0.24; ethnicity, *p =* 0.70) or BMI (*p =* 1.00) of the recipients.

**Table 2:** Metabolic parameters in FMT and placebo groups throughout the 12-week study.

Characteristic	Placebo group			FMT group			Difference between FMT and placebo groups in change from baseline (95% CI)	
	Baseline	6 weeks	12 weeks	Baseline	6 weeks	12 weeks	Baseline to 6 weeks	Baseline to 12 weeks
Weight (kg)	111 ± 20	111 ± 20	111 ± 19	110 ± 26	114 ± 26	111 ± 27	−0.2 (−2.4, 2.0)	0.2 (−2.0, 2.4)
Lean mass (kg)	58 ± 12	58 ± 12	58 ± 11	60 ± 15	62 ± 15	61 ± 16	−0.4 (−2.1, 1.4)	−0.1 (−1.9, 1.6)
Fat mass (kg)	53 ± 10	53 ± 10	52 ± 10	49 ± 13	51 ± 14	50 ± 14	1.1 (−0.7, 3.0)	1.2 (−0.6, 3.0)
VAT volume (cm^3^)	998 ± 319	991 ± 285	976 ± 308	1048 ± 368	1107 ± 423	982 ± 358	19 (−76, 115)	−52 (−147, 42)
Fasting glucose (mmol/l)	4.8 ± 0.4	4.8 ± 0.4	5.1 ± 0.6	5.0 ± 0.7	4.8 ± 0.7	5.1 ± 0.6	0.02 (−0.3, 0.4)	−0.1 (−0.4, 0.3)
HbA1c (%)	5.5 ± 0.3	5.5 ± 0.3	5.5 ± 0.3	5.6 ± 0.2	5.5 ± 0.4	5.4 ± 0.4	−0.1 (−0.2, 0.1)	**−0.1 (−0.3, −0.01)**
HOMA-IR	3.5 ± 1.9	3.4 ± 1.3	4.8 ± 1.7	3.5 ± 1.4	3.9 ± 1.4	4.7 ± 2.0	0.3 (−0.6, 1.3)	−0.02 (−0.9, 0.9)
Total cholesterol (mmol/l)	5.1 ± 0.6	5.1 ± 1.1	5.2 ± 0.7	5.5 ± 0.6	5.2 ± 0.8	5.2 ± 1.0	−0.3 (−0.8, 0.2)	−0.3 (−0.8, 0.2)
HDL (mmol/l)	1.2 ± 0.3	1.1 ± 0.3	1.1 ± 0.4	1.3 ± 0.4	1.3 ± 0.5	1.3 ± 0.3	0.04 (−0.1, 0.2)	0.08 (−0.1, 0.2)
LDL (mmol/l)	3.3 ± 0.6	3.3 ± 1.2	3.2 ± 0.7	3.3 ± 0.8	3.0 ± 0.9	2.9 ± 0.9	−0.2 (−0.6, 0.2)	−0.2 (−0.6, 0.2)
Triglycerides (mmol/l)	1.3 [1.1, 1.8]	1.2 [1.1, 2.0]	1.4 [1.0, 2.7]	1.7 [1.1, 2.2]	1.9 [1.2, 2.3]	1.5 [1.3, 2.1]	−0.4 (−1.4, 0.5)	−0.8 (−1.7, 0.1)
CRP (mg/l)	3.5 [2.3, 7.3]	3.0 [1.7, 5.0]	4.6 [2.5, 6.8]	2.9 [1.7, 5.6]	3.5 [1.9, 5.0]	2.9 [2.0, 4.1]	**1.8 (0.3, 3.3)**	−0.1 (−1.6, 1.3)
REE (kcal/day)*	1,503 ± 218	1,536 ± 241	n/a	1,588 ± 305	1,705 ± 351	n/a	8.4 (−97, 114)	n/a
Caloric intake (kcal/day)	1,939 ± 463	2,006 ± 693	1,689 ± 760	2,121 ± 729	2,236 ± 949	2,331 ± 822	−50 (−603, 502)	389 (−155, 932)

Data are mean ± SD or median [Q1, Q3]. Mean differences between FMT and placebo groups with 95% confidence intervals were calculated for change between baseline and 6 or 12 weeks using longitudinal mixed effects modeling. Bold font indicates statistically significant differences between the FMT and placebo groups.

*REE was not measured at the 12-week study visit.

CRP, C-reactive protein; FMT, fecal microbiota transplantation; HbA1c, hemoglobin A1c; HDL, high-density lipoprotein; HOMA-IR, homeostatic model assessment of insulin resistance; LDL, low-density lipoprotein; n/a, not available; REE, resting energy expenditure; VAT, visceral adipose tissue.

**Fig 3:**
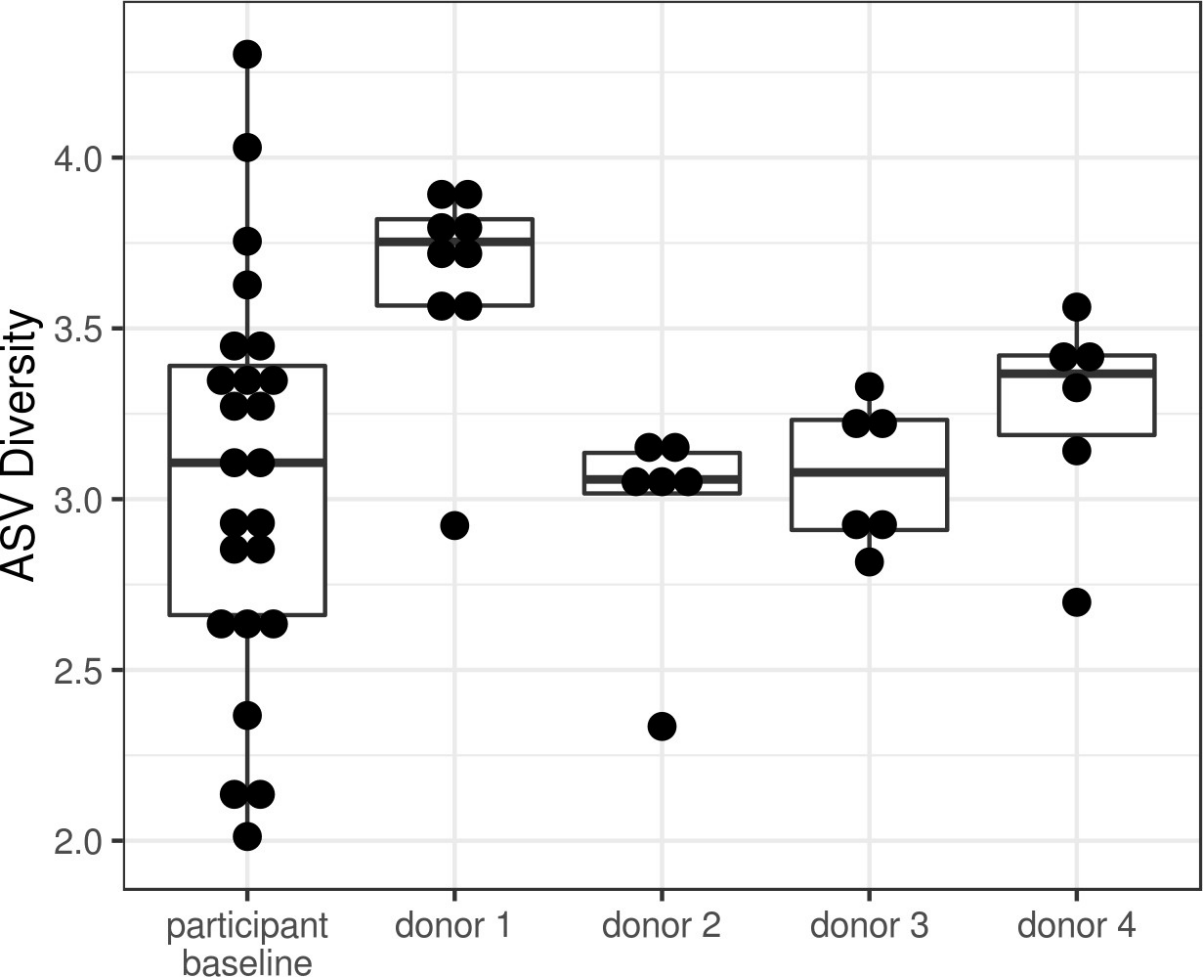
Boxplot displaying the amplicon sequence variant (ASV) diversity (Shannon diversity index) identified in lean donor samples and baseline samples of participants with obesity.

Baseline microbiome analysis revealed that, with the exception of donor 1, preps from the same donor tended to cluster separately from baseline samples of participants with obesity (Fig B in S1 Data). Preps from donor 1 demonstrated distinctively high diversity, with approximately 50% more unique 16S V4 DNA sequences (ASVs) than preps from the other 3 donors (Fig 3), whereas the median diversity of the preps from the other 3 donors fell within the interquartile range of the baseline participant samples.

**Fig 4:**
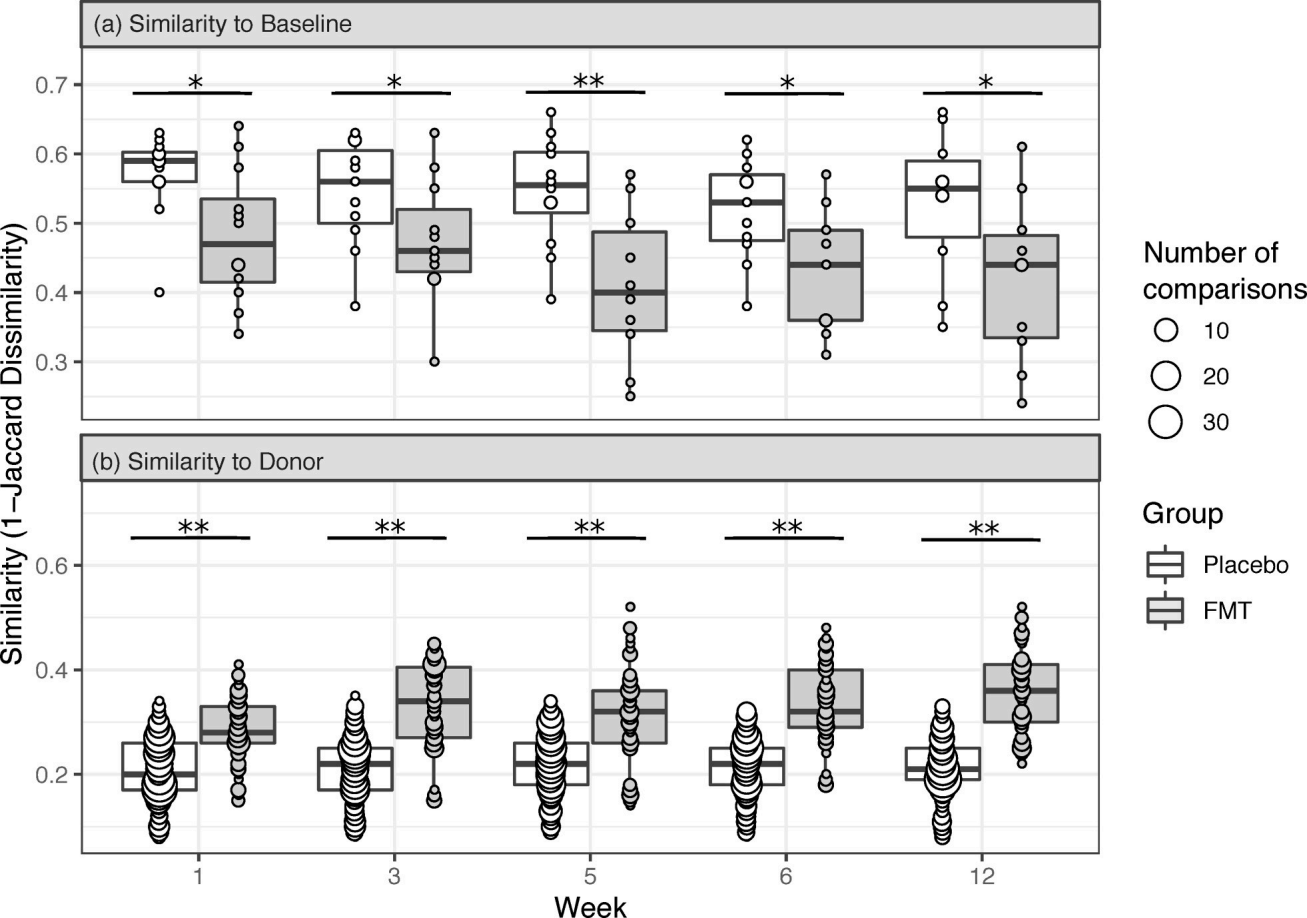
Beta diversity boxplots displaying microbiome compositional similarity of each participant to their respective baseline or triplicate donor preps. Microbiome similarity to baseline (a) and to donor (b) is compared between fecal microbiota transplantation (FMT) and placebo groups. Placebo results shown in (b) reflect comparisons of all combinations of placebo participant to donor prep samples. However, for Wilcoxon rank sum tests comparing similarities between FMT and placebo recipients, the similarity of each placebo participant sample to all donor prep samples was first averaged. *p < 0.05; **p < 0.01.

As expected, we observed temporal variability in placebo participant microbiomes, as well as some background similarity between placebo samples and donor material (Fig 4). We thus used the variability in placebo participant microbiome data to define the background level of endogenous microbiome variation in our analyses. The microbiomes of FMT recipients following dosing were more similar in composition to their paired donor material and less similar in composition to their own baseline sample, compared to placebo participants (Fig 4). These data suggest that FMT shifted recipients’ microbiomes away from their respective baseline compositions and towards donor compositions. Subdividing FMT recipients by their donor material revealed that this shift was observed among recipients of FMT from donors 1, 3, and 4 (Fig C in S1 Data). Although the microbiome of the single donor 2 FMT recipient did not exhibit a large shift towards the microbiome composition of donor 2 (Fig C in S1 Data, panel A), this recipient’s microbiome did demonstrate a potential shift away from baseline following FMT (Fig C in S1 Data, panel B).

**Fig 5:**
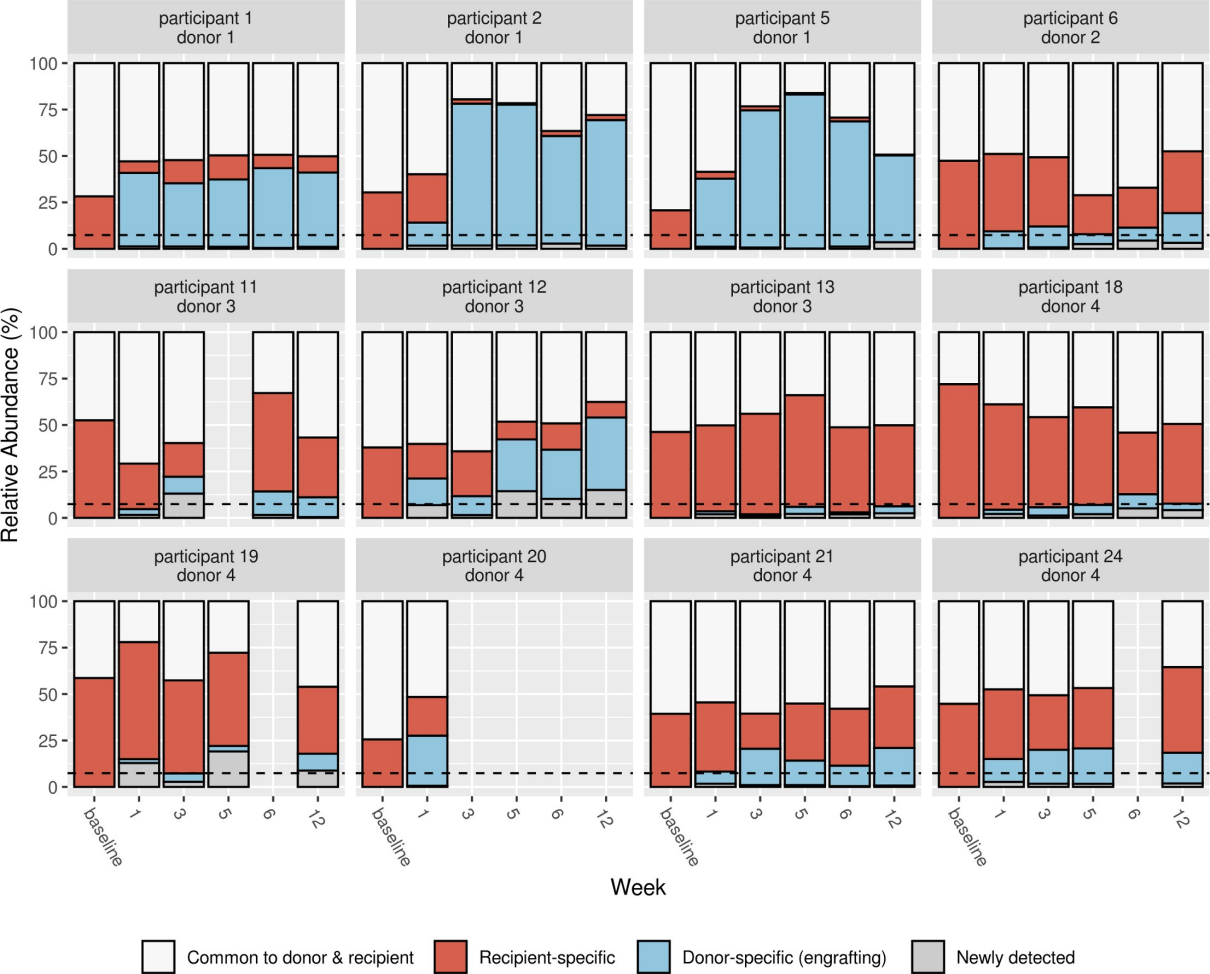
Proportion of bacterial amplicon sequence variants (ASVs) for each fecal microbiota transplantation (FMT) participant and timepoint hypothesized to originate from the participant’s or donor’s microbiome. Each facet is labeled by participant (first line) and paired donor (second line). Red bars indicate ASVs present in participant baseline and follow-up samples, and thus thought to be native to the participant. ASVs originating from the donor and not detected in paired participant baseline samples, and thus thought to be engrafting ASVs, are shown in blue. ASVs only observed following treatment and not seen in paired donor material are categorized as newly detected and displayed in gray. ASVs shown in white were observed in both paired donor and baseline recipient samples, and thus we were unable to resolve whether the ASVs at post-dosing timepoints came from strains native to the participant or donor material. The 75th quartile of newly detected ASVs across post-dosing placebo participant samples is delineated by a dotted line for comparison.

We further defined specific 16S V4 DNA sequences as donor-specific (engrafting) ASVs if they were present in both donor material and post-dosing recipient samples but not in baseline recipient samples for donor–recipient pairs. ASVs observed in post-baseline recipient samples but not observed in paired sequenced donor material were categorized as newly detected, not engrafting. All FMT recipients exhibited engraftment of donor-specific ASVs (Fig 5), but the relative abundances of the engrafting ASVs were highly variable. For example, the percentage of total reads in post-dosing recipient microbiome samples that mapped to engrafting ASVs was notably high among donor 1 FMT recipients, with a sample median of 47%, whereas median abundances for the other donors were around 9.5%. In the majority of FMT recipients, total abundance of engrafting ASVs exceeded the background level of newly detected ASVs (Fig 5). The exceptions were participants 13 and 18, for whom the majority of post-dosing samples suggested that the rate of newly detected and engrafting ASVs did not exceed the expected background variation [33].

Overall, our data suggest that bacterial strains from donor FMT capsules successfully engrafted in the majority of our participants, although engraftment was markedly strongest among donor 1 FMT recipients and may not have occurred at all in participants 13 and 18. When occurring, engraftment persisted throughout the 12-week study, including 6 weeks after the cessation of dosing. Participant microbiome similarity to donor material plateaued after week 3, suggesting that dosing for more than 3 weeks does not result in additional engraftment. However, the additional weeks of dosing could have contributed to maintenance of engraftment.

The engrafting ASVs from all donors represented a diverse set of enteric genera (Fig D–G in S1 Data). Strikingly, we observed that 2 *Prevotella* ASVs strongly engrafted in donor 1 FMT recipients, increasing the relative abundance of Prevotellaceae in donor 1 FMT recipient microbiomes by at least 8-fold post-dosing and thus shifting the enterotype signature of these participants towards a higher *Prevotella* abundance relative to *Bacteroides* (*P/B* ratio) (Fig H in S1 Data). No enterotype shifts were consistently observed among other recipients. Despite the evidence for engraftment, compared to placebo participants, FMT-treated participants did not display a notable increase in microbiome diversity from baseline to week 6, suggesting that the FMT treatment did not impact participant microbiome diversity (Fig I in S1 Data).

A large percentage of ASVs were identified in both donor and baseline recipient material. It is unclear whether those ASVs represent the same bacterial strain present in both samples or whether the donor and participant microbiomes each contained different bacterial strains that could not be differentiated due to the limited taxonomic resolution provided by 16S V4 sequencing. To address this, we sequenced a subset of donor 1 FMT recipient and placebo samples using shotgun metagenomics, which can provide finer taxonomic resolution. These data recapitulated ASV engraftment results, showing that the 3 donor 1 FMT recipients had more bacterial species in common with donor material, and fewer bacterial species in common with their own baseline sample relative to the 3 placebo participants (Fig J in S1 Data). Additionally, among the bacterial species found in both baseline and post-FMT shotgun metagenomic samples, approximately 50% the bacterial strains of those species identified in weeks 6 and 12 were more phylogenetically related to strains found in the donor material than the baseline recipient sample (Fig K in S1 Data), suggesting those organisms originated from the FMT dose.

We did not observe any statistically significant correlations between changes in participant microbiome composition and changes in metabolic outcomes. Given prior studies that have shown low bacterial diversity as predictive of greater metabolic response to FMT [24], we performed exploratory subset analyses. Among participants with low baseline microbiome diversity (Table B in S1 Data), analyses suggested greater improvements in several metabolic outcomes at 12 weeks for those who received FMT (*n =* 4) versus placebo (*n =* 7), including total cholesterol (mean difference −0.6 mmol/l, 95% CI −1.0 to −0.1 mmol/l), HbA1c (mean difference −0.2%, 95% CI −0.4 to −0.01%), and fasting glucose (mean difference −0.6 mmol/l, 95% CI −1.1 to −0.1 mmol/l).

There were no serious AEs reported in either group throughout the 12-week study (Table C in S1 Data). More study participants reported at least 1 episode of diarrhea in the FMT group than in the placebo group, although this difference was not statistically significant (10 versus 5, *p =* 0.09). The majority of diarrheal symptoms were rated as mild, and there were no CTCAE grade 3+ AEs. Intriguingly, the only 4 diarrheal events rated as moderate occurred in 2 participants who received FMT from donor 4, one of whom dropped out of the study following their second event. Upon inspection of engrafting ASVs, no organisms stood out as the potential cause of the moderate AEs (Fig F in S1 Data) albeit several days separated the timing of events and stool collection. There were no imbalances between FMT and placebo groups in other symptoms.

## Discussion

This double-blind randomized placebo-controlled trial was designed to test the safety of FMT capsules and their ability to alter the gut microbiome in adults with obesity, and to probe for a causal link between microbial changes and metabolism. We found that 6 weeks of FMT capsule administrations sustainably altered gut microbiome composition for the majority of participants without serious adverse effects, and absent any antibiotic pretreatment or lavage. Despite the encouraging engraftment signal, we did not find statistically significant differences between the FMT and placebo groups in insulin resistance, body weight, or most other metabolic markers in these adults with obesity and without diabetes. HbA1c modestly decreased at 12 weeks in the FMT group as compared to the placebo group, although the magnitude of improvement was small. Both metabolic and microbiome responses to FMT were highly variable, suggesting a complex host–recipient dynamic.

To date, only Nieuwdorp and colleagues have published interventional FMT trials in patients with obesity. In a small pilot trial, conducted in the Netherlands, 9 men with obesity who received endoscopically delivered FMT infusions from lean donors had significantly improved peripheral insulin sensitivity over 6 weeks as assessed by hyperinsulinemic euglycemic clamp [23]. The median level of insulin-mediated glucose uptake was approximately 73% higher at 6 weeks than at baseline, and was accompanied by increased gut microbial diversity. A larger follow-up study by the same group once again demonstrated a statistically significant increase in peripheral insulin sensitivity among 26 adults with obesity receiving endoscopic FMT infusion from lean donors, although the magnitude of improvement was more modest (approximately 12%) [24]. This follow-up study also documented a small decline in HbA1c at 6 weeks after FMT, which is similar to the minor improvement in HbA1c at 12 weeks in our trial. However, engrafting clades were different in the 2 prior studies, and the studies also documented different patterns of change in fecal short chain fatty acids and bile acids. In both prior studies, metabolic and microbiome changes were short-lived, having disappeared by 12–18 weeks after FMT infusion [23,24].

The aforementioned small studies as well as our current pilot trial are not definitive and should be considered hypothesis-generating. Indeed, one possible interpretation of our study is that the gut microbiome does not regulate human metabolism in the same dramatic manner as has been shown in preclinical studies. Alternatively, it is possible that a greater magnitude of FMT engraftment is required to effect systemic changes. Mouse models have shown that obesity phenotypes can be transferred to germ-free mice through colonization with obese mouse microbiota [4,5]. However, there is little evidence to date informing whether obese or lean phenotypes can be transferred to already colonized models or the extent to which the native microbiome needs to be replaced to induce a phenotypic change.

The closest human equivalent to colonizing a germ-free mouse would be the total replacement of an individual’s native microbiome, which to our knowledge has never been demonstrated. Strategies have been proposed to further improve FMT engraftment in clinical studies. For example, treating our study participants with broad-spectrum antibiotics prior to FMT dosing would likely have increased the ratio of donor to baseline microbes after FMT [34]. However, this approach could be associated with side effects and raise ethical and antibiotic stewardship concerns. It has been suggested that bowel cleansing might enhance FMT engraftment, but this strategy has not been rigorously studied, and bowel preps only minimally impact microbiome composition [35]. For these reasons, combined with the absence of a clear experimentally supported engraftment level target, we elected to begin testing the impact of FMTs on metabolic outcomes in human participants with the most minimally invasive approach. Indeed, we found evidence of engraftment in the majority of the FMT recipients without any gut preparation or pretreatment antibiotics. It nevertheless remains unclear if achieving a greater number or relative abundance of engrafting strains in our clinical study would have yielded positive metabolic outcomes more akin to results from germ-free mouse models.

It is helpful to contrast our pilot trial with the prior published clinical trials of FMT and metabolism. Baseline age, BMI, and HbA1c in our study were roughly comparable to those of participants in the Netherlands projects. However, microbiomes of donors and recipients can strongly vary in diversity, composition, and engraftment strength, as is evident in our study data, and not all donor material may be equally efficacious, nor all recipient microbiomes equally responsive to a microbiome therapy. Notably, our trial was conducted in the US, and we expect the recipients and donors from the US and Netherlands studies to have different microbiome compositions due to regional, race/ethnicity, and dietary differences in the study populations [36–38]. Furthermore, Nieuwdorp and colleagues enrolled men exclusively, whereas our recipients receiving active FMT treatment were 67% women, and 3 out of our 4 donors were women. There are some data to suggest differences in the gut microbiome between men and women [39], and thus it is possible that there are sex-specific differences in donor FMT material, or altered metabolic responses to FMT among female recipients. The routes of FMT delivery (capsule versus endoscopy), type of FMT (frozen versus fresh), choice of controls (non-microbiome placebo versus autologous FMT), and antecedent gastrointestinal preparation (none versus bowel prep) differed from prior studies. These have not been important factors in studies of FMT for recurrent *C*. *difficile* colitis [40], although appropriate caution should be taken when extrapolating from FMT outcomes in other disease conditions.

Underlying causes of obesity and insulin resistance are multifactorial and likely vary among individuals. It is possible that only a subset of individuals may respond to alterations of the microbiome. Of note, the second Netherlands study reported that lower gut microbiota diversity among obese recipients at baseline predicted metabolic response to FMT [24]. In our exploratory analyses, we observed that FMT capsules led to possible improvements in total cholesterol, fasting glucose, and HbA1c among those with low microbiome diversity at baseline, although these results should be interpreted with caution given the small numbers of participants studied. It is also important to note that the magnitude of clinical improvement after FMT in these exploratory studies is modest. We found that donor microbial diversity was positively associated with better engraftment, although changes in microbial composition were not specifically correlated with metabolic outcomes. We used a different method for characterizing microbial communities than that used in the Netherlands studies; thus, direct comparisons between cohorts are difficult to make. Variable engraftment by donor and variable metabolic changes among FMT recipients raise the question of whether selecting donors with specific microbiome signatures or designing a targeted doseable microbial consortium could yield metabolic improvements.

In a noteworthy study, gut microbiome composition helped to predict glycemic responses to various diets [41]. A specific enterotype defined by a high abundance of *Prevotella* or a high ratio of *Prevotella* relative to *Bacteroides* (*P/B* ratio) [42] has also shown promise in predicting response to dietary interventions. For example, Kovatcheva-Datchary et al. observed that participants with a high abundance of *Prevotella* experienced greater improvements in glucose metabolism following fiber intervention than participants with low *Prevotella* abundance [43]. In addition, a study by Hjorth et al. found that participants with *P/B* ratios above 0.01 lost more weight after switching to a high-fiber healthy diet than participants with a *P/B* ratio below that threshold [44]. Thus, it is possible that additional selection criteria may be required to produce a microbiome therapeutic to optimize engraftment and metabolic responses, perhaps in the setting of a concurrent dietary intervention.

### Strengths and limitations

Strengths of our study include the use of hyperinsulinemic euglycemic clamps, the gold-standard assessment of insulin sensitivity. In addition, we used a rigorous randomized study design with placebo controls and masking of group assignments. Limitations of this study include the small sample size of this pilot trial and the heterogeneous study population, and inclusion of participants with only mild insulin resistance, perhaps hampering our ability to detect improvements in insulin sensitivity. Additionally, we did not assess hepatic insulin sensitivity based upon prior studies that only found FMT effects on peripheral insulin resistance [23,24]. Although we used sophisticated microbiome analysis tools, we were mostly limited to the taxonomic resolution available from the 16S V4 region, such that roughly 25%–75% of reads in each participant’s baseline sample could not be differentiated from donor sequences. Nevertheless, results from shotgun sequencing performed on a subset of samples were consistent with our 16S V4 data suggesting engraftment. As noted earlier, metabolic and microbiome responses were highly variable, and our study was not sufficiently powered to characterize subgroups of responders or outcomes specific to individual donors. Finally, we did not introduce a dietary intervention to this study design, and our study participants consumed typical high-fat, low-fiber “Western” diets. Given that a previous study in germ-free mice found that the obese or lean phenotype was only transmissible via FMT in the presence of a low-fat, high-fiber diet [3], it is possible that pairing gut microbiota modulation with a dietary intervention may be required to enhance metabolic response.

### Conclusion

Repeated weekly oral FMT by an encapsulated frozen inoculum is safe and tolerable in adults with obesity, and oral FMT without “conditioning” the gut with antibiotics or a bowel cleanse can result in gut microbiota engraftment for at least 12 weeks in the majority of recipients. Despite the engraftment signal, we did not observe statistically significant changes in insulin sensitivity or most other metabolic parameters. Thus, it seems unlikely that FMT-induced microbiome compositional changes alone will be sufficient to treat or prevent metabolic disorders in humans. Future research should explore whether pre-selection of donors and/or recipients or specifically designed microbial compositions can optimize beneficial microbiota changes, and whether use of a microbiome intervention in conjunction with a dietary/exercise intervention may lead to synergistic metabolic improvements in adults with obesity.

## Supporting information

S1 CONSORT Checklist Completed CONSORT checklist. (DOC)

S1 Data Supplementary tables and figures. (PDF)

S1 Methods Supplementary methods. (DOCX)

S1 Obesity FMT Protocol Clinical trial protocol. (DOCX)

## Abbreviations

AE: adverse event
ASV: amplicon sequence variant
CTCAE: Common Terminology Criteria for Adverse Events
DXA: dual-energy X-ray absorptiometry
FMT: fecal microbiota transplantation
HOMA-IR: homeostatic model assessment of insulin resistance
