# Supplementary material for: Fecal microbiota transplantation for the improvement of metabolism in obesity: The FMT-TRIM double-blind placebo-controlled pilot trial
Source: PLoS Med. 2020 Mar 9;17(3):e1003051. doi: 10.1371/journal.pmed.1003051 (PMC7062239; doi:10.1371/journal.pmed.1003051)

Supplementary Table A. Baseline characteristics of FMT donors.

Donor	Age	Sex	Race/ Ethnicity	Height (cm)	Weight (kg)	BMI (kg/m2)	HbA1c (%)	Fasting glucose (mmol/L)	Dietary overview
**#1**	18	F	Caucasian	163.8	52.4	19.5	4.6	5.3	omnivore, often vegan
**#2**	22	F	Caucasian	172.7	60.1	20.1	5.0	4.3	vegetarian for 12 years
**#3**	24	F	Caucasian	165.1	59.4	21.8	5.2	5.3	omnivore
**#4**	37	M	Caucasian	188.0	172.6	20.5	5.2	4.9	avoids processed foods

Supplementary Table B. Metabolic parameters in subset of FMT and placebo recipients with low baseline microbial diversity. Due to small sample size, all data are presented as median [range]. *REE and insulin-mediated glucose uptake (M) were not measured at the 12-week study visit. P-values indicate time x group interaction as a comparison of change in each metabolic parameter between FMT and placebo groups within this subset using longitudinal mixed effects modeling. Bold font indicates statistically significant differences between FMT and placebo groups.

	**Placebo capsules N = 7**			**FMT capsules N = 4**			**Difference between FMT and placebo in change from baseline (95% Confidence Interval)**	
	Baseline	6 weeks	12 weeks	Baseline	6 weeks	12 weeks	Baseline to 6 weeks	Baseline to 12 weeks
**Weight (kg)**	105 [78-150]	105 [78-150]	107 [80-148]	87 [77-122]	90 [85-120]	87 [78-121]	-0.1 (-2.3,2.1)	0.8 (-2.8,1.3)
**Lean Mass (kg)**	53 [40-78]	54 [41-78]	53 [39-73]	49 [42-57]	52 [48-55]	50 [43-56]	-0.3 (-3.9,3.3)	0.1 (-3.4,3.5)
**Fat Mass (kg)**	52 [37-69]	52 [36-69]	51 [41-71]	37 [34-64]	37 [37-66]	37 [35-66]	1.2 (-1.6,4.0)	1.5 (-1.2,4.1)
**VAT Volume (cm^3)**	938 [437-1242]	876 [499-1344]	847 [536-1233]	810 [649-897]	874 [657-1048]	731 [655-810]	3 (-162,168)	-30 (-188,127)
**Fasting glucose (mmol/L)**	4.7 [3.9-5.1]	4.4 [4.4-5.7]	4.8 [4.5-6.2]	4.6 [4.1-6.2]	4.2 [3.9-4.9]	4.6 [4.2-5.7]	-0.2 (-0.7,0.3)	**-0.6 (-1.1,-0.1)**
**HbA1c (%)**	5.4 [5.0-5.8]	5.5 [5.0-5.7]	5.4 [5.0-5.8]	5.5 [5.3-5.8]	5.4 [5.4-5.4]	5.3 [5.0-5.8]	-0.02 (-0.2,0.20)	**-0.22 (-0.42,-0.01)**
**HOMA-IR**	3.3 [0.8-8.6]	3.5 [2.2-5.9]	4.7 [3.5-7.7]	3.5 [1.7-4.8]	4.2 [3.9-6.4]	4.5 [3.9-5.6]	1.5 (-1.2,4.2)	-0.2 (-2.7,2.4)
**Total Cholesterol (mmol/L)**	4.9 [4.4-5.1]	4.7 [4.2-5.2]	4.9 [4.0-5.3]	5.6 [5.0-6.1]	4.8 [4.6-5.6]	4.9 [4.6-5.7]	**-0.5 (-1.0,-0.1)**	**-0.6 (-1.0,-0.1)**
**HDL (mmol/L)**	1.2 [0.7-1.4]	1.2 [0.7-1.4]	1.2 [0.7-1.6]	1.2 [0.8-1.4]	1.4 [0.8-1.3]	1.3 [1.0-1.4]	0.1 [-0.2,0.4)	0.1 (-0.2,0.4)
**LDL (mmol/L)**	2.9 [2.7-3.5]	2.7 [2.4-3.4]	2.8 [2.3-3.6]	3.8 [3.0-4.0]	3.3 [2.0-3.5]	2.9 [2.0-3.8]	-0.3 (-0.8,0.3)	-0.3 (-0.8,0.2)
**Triglycerides (mmol/L)**	1.3 [0.7-2.6]	1.1 [0.9-2.9]	1.1 [0.8-3.5]	1.8 [1.5-8.5]	1.9 [1.6-3.1]	1.8 [1.3-3.2]	-1.8 (-3.8,0.1)	-1.6 (-3.5,0.2)
**CRP (mg/L)**	3.5 [0.3-14.8]	3.2 [0.3-18.5]	4.2 [0.3-18.7]	4.4 [1.5-5.7]	5.0 [3.1-6.8]	4.1 [2.9-7.9]	2.1 (-0.6,4.7)	-0.3 (-2.9,2.2)
**REE (kCal/day)***	1456 [1149-1818]	1412 [1215-1802]	n/a	1621 [1428-1633]	1642 [1335-1671]	n/a	-6.8 (-102,88)	n/a
**Caloric intake (kCal/day)**	2056 [1277,2895]	1636 [1133,2694]	1225 [538,2220]	1865 [1362-2090]	2338 [1142-2384]	1899 [1686-2154]	430 (-229,1089)	649 (27,1271)
**M (mg/kg/min)***	9.6 [3.4-10.8]	9.2 [2.7-10.4]	n/a	6.8 [5.7-10.1]	6.2 [5.9-6.8]	n/a	0.6 (-1.6,2.8)	n/a

Supplementary Table C. Adverse events in FMT and placebo groups throughout the 12-week study. All symptoms noted below are grade 1 or 2 unless otherwise noted.

Symptoms	Placebo	FMT	P-Value
**Fever**	2	1	1.00
**Diarrhea**	5	10	0.09
**Nausea/Vomiting**	6	6	1.00
**Fatigue/Malaise**	3	5	0.67
**Headache**	4	5	1.00
**Distension/Bloating, Abdominal pain/Discomfort**	5	7	0.68
**Any moderate or severe adverse events, grade 3 or 4**	0	0	1.00

Supplementary Figure A. Boxplot of percentage change in normalized insulin sensitivity in FMT and placebo groups from baseline to 6 weeks. Insulin stimulated glucose uptake (M) was assessed by hyperinsulinemic euglycemic clamp and then corrected for steady-state insulin level (M/I).

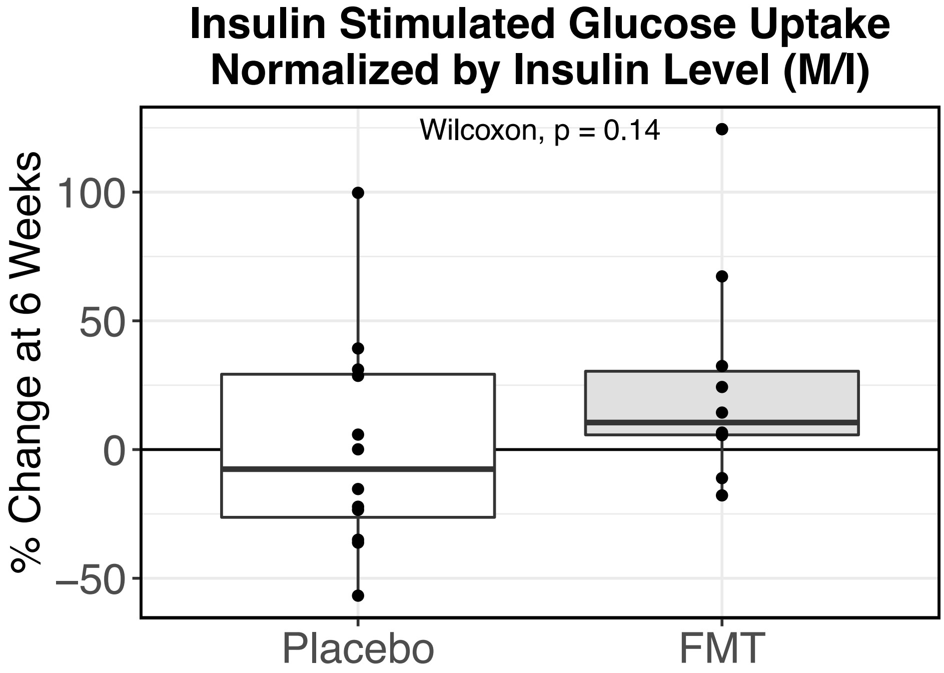

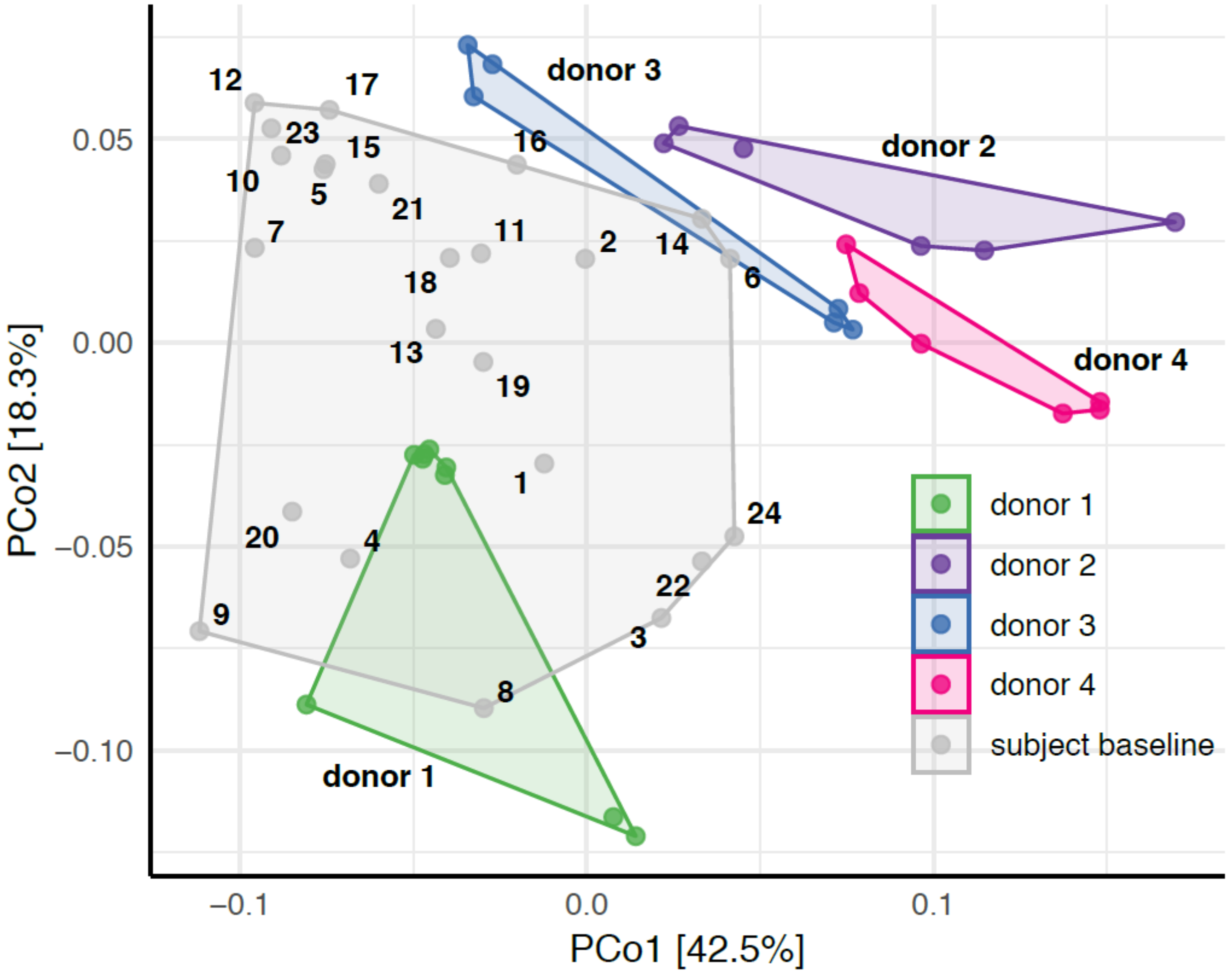

Supplementary Figure B. Principal Coordinates Analysis (PCoA) based on weighted unifrac displaying microbiome similarities between lean donor preps and obese subject baseline samples. Preps from the same donor and all subject's baseline samples are outlined by polygons.

Supplementary Figure C. Beta diversity boxplots displaying microbiome compositional similarity of each subject to triplicate donor preps (A) or their respective baseline (B), separated by donor material. Microbiome similarity is compared between FMT and placebo groups. Placebo results shown in panel A reflect comparisons between all combinations of placebo subjects to all donor prep samples.

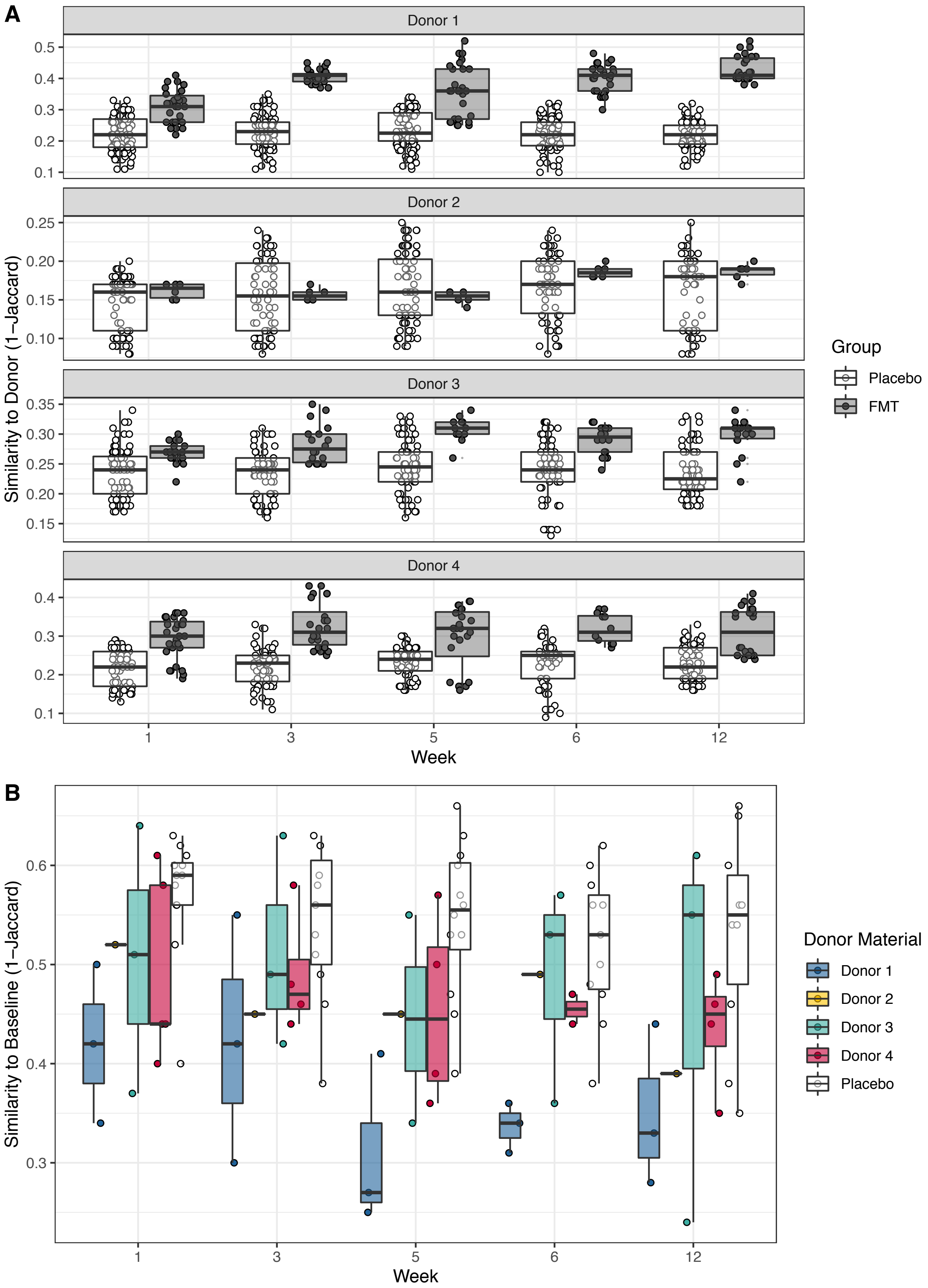

Supplementary Figure D. Single heatmap displaying the relative abundance of putative engrafting ASVs across Donor 1 preps and paired recipient samples. ASVs are ordered based on similarity of abundance profiles across subject samples. Three subjects received FMT capsules generated from Donor 1.

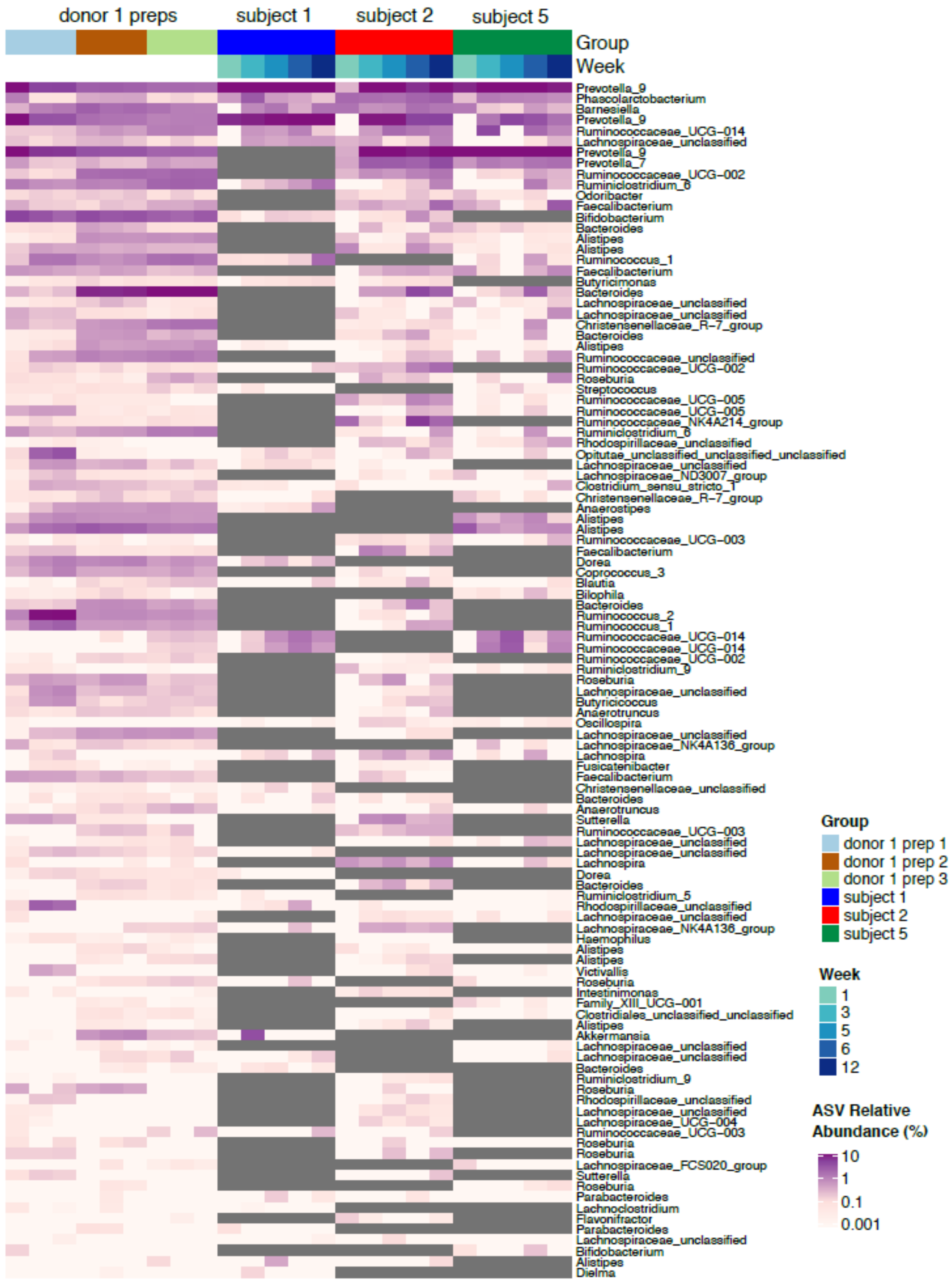

Supplementary Figure E. Single heatmap displaying the relative abundance of putative engrafting ASVs across Donor 2 preps and paired recipient samples. ASVs are ordered based on similarity of abundance profiles across subject samples. One subject received FMT capsules generated from Donor 2.

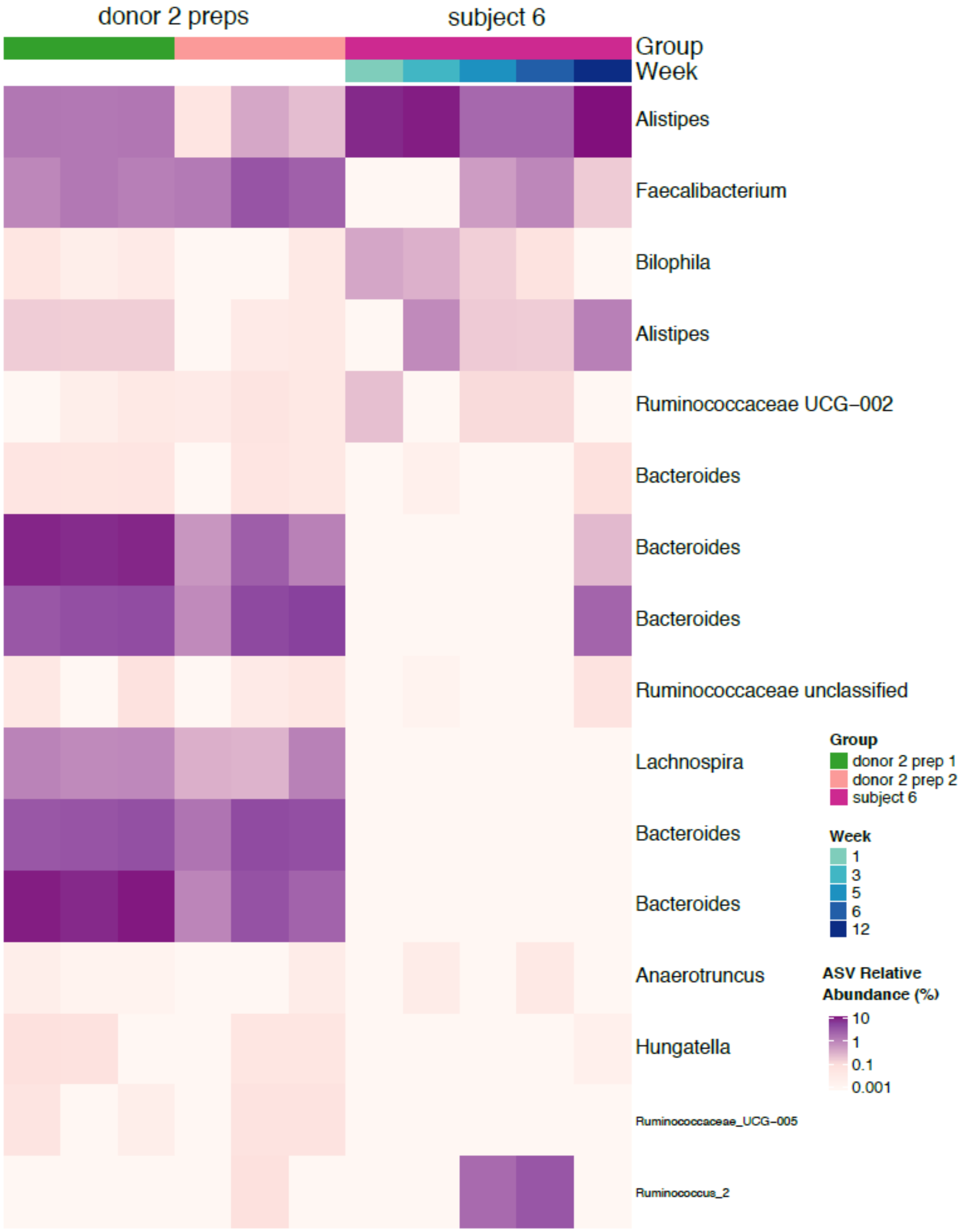

Supplementary Figure F. Single heatmap displaying the relative abundance of putative engrafting ASVs across Donor 3 preps and paired recipient samples. ASVs are ordered based on similarity of abundance profiles across subject samples. Three subjects received FMT capsules generated from Donor 3.

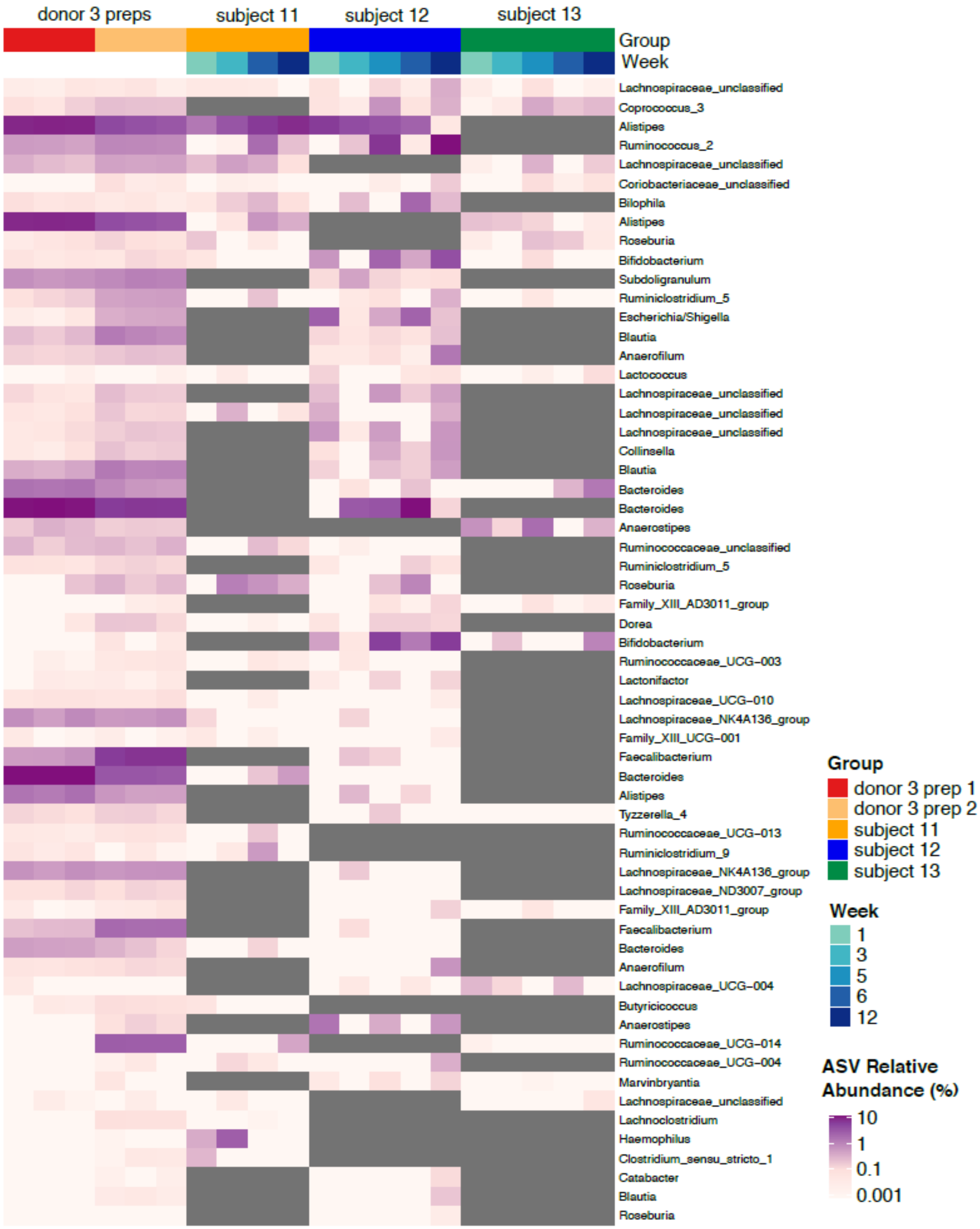

Supplementary Figure G. Single heatmap displaying the relative abundance of putative engrafting ASVs across Donor 4 preps and paired recipient samples. ASVs are ordered based on similarity of abundance profiles across subject samples. Five subjects received FMT capsules generated from Donor 4.

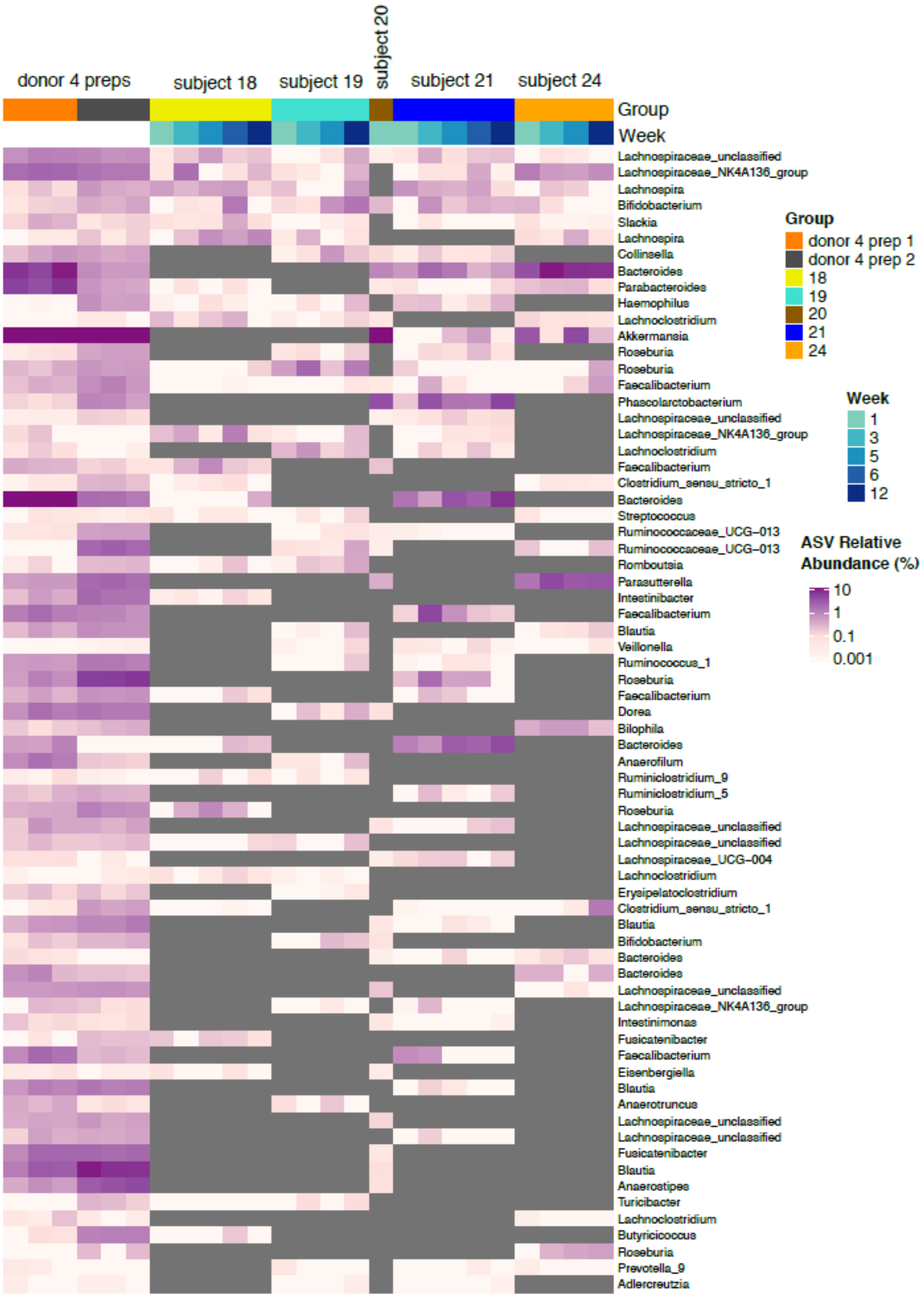

Supplementary Figure H. The ratio of *Prevotellaceae* to *Bacteroidaceae* abundances in individual subjects throughout the study. Material from each donor and paired recipient subject, or all Placebo subjects are plotted in the same facet. Gray dots mark the *P/B* ratio of all donor preps and each line tracks the change in *P/B* ratios of a subject across the study's time course. The Hjorth et al. *P/B* threshold represents the value above which predicted greater weight loss during a dietary intervention study.[1]

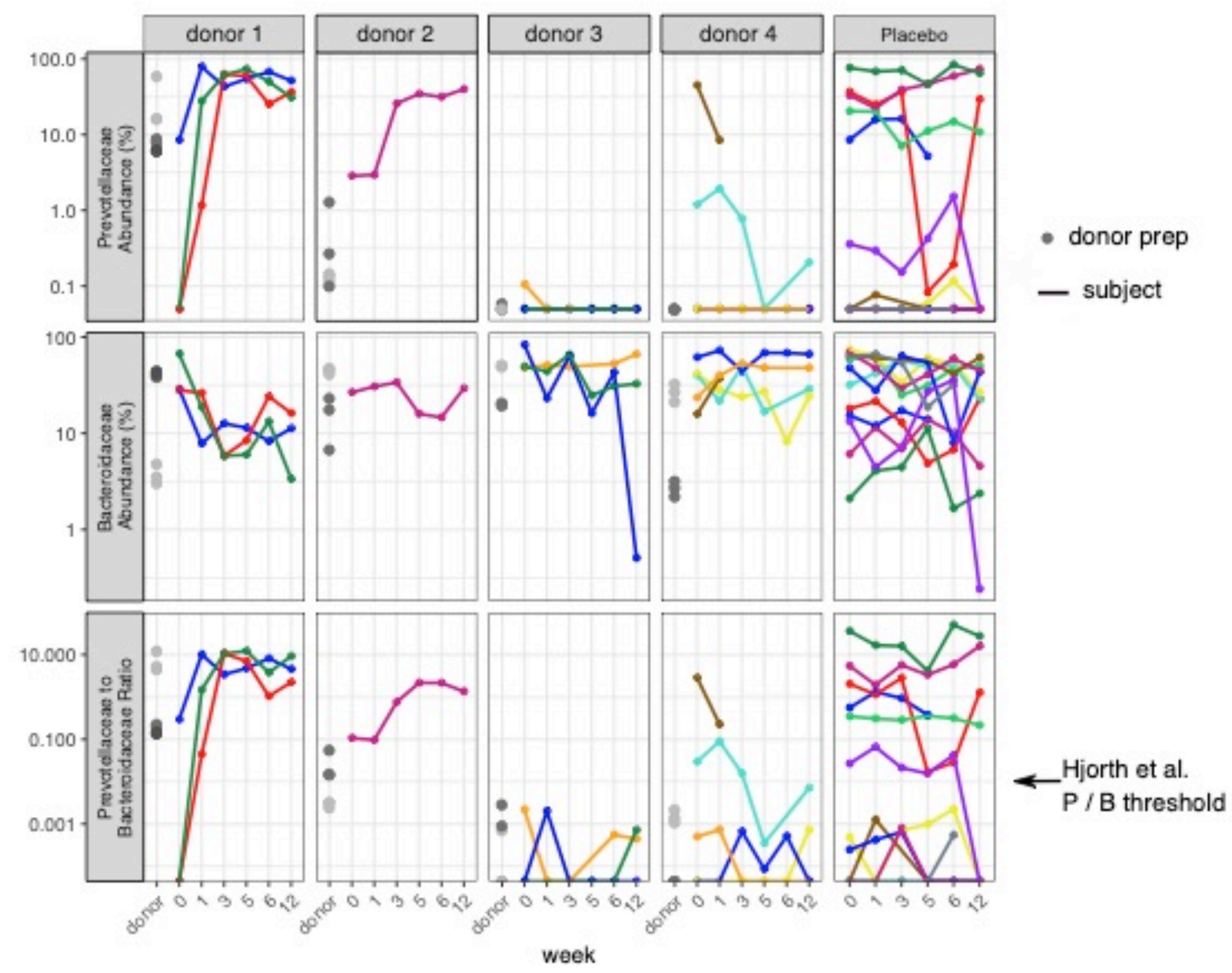

Reference:

1. Hjorth MF, Roager HM, Larsen TM, et al. Pre-treatment microbial Prevotella-to-Bacteroides ratio, determines body fat loss success during a 6-month randomized controlled diet intervention. *Int J Obes (Lond)* 2018; **42**(3): 580-3.

Supplementary Figure I. Change in microbiome richness (a) and diversity (b) across dosing period. Lines connect samples from the same subject and line type reflects received dose material. Percent change from week 0 to week 6 was not statistically different between FMT and placebo groups (Wilcoxon Rank Sum test p > 0.05). Three subject week 6 microbiome samples yielded low sequence counts, so week 5 samples were used instead to maintain consistency in data quality across this analysis.

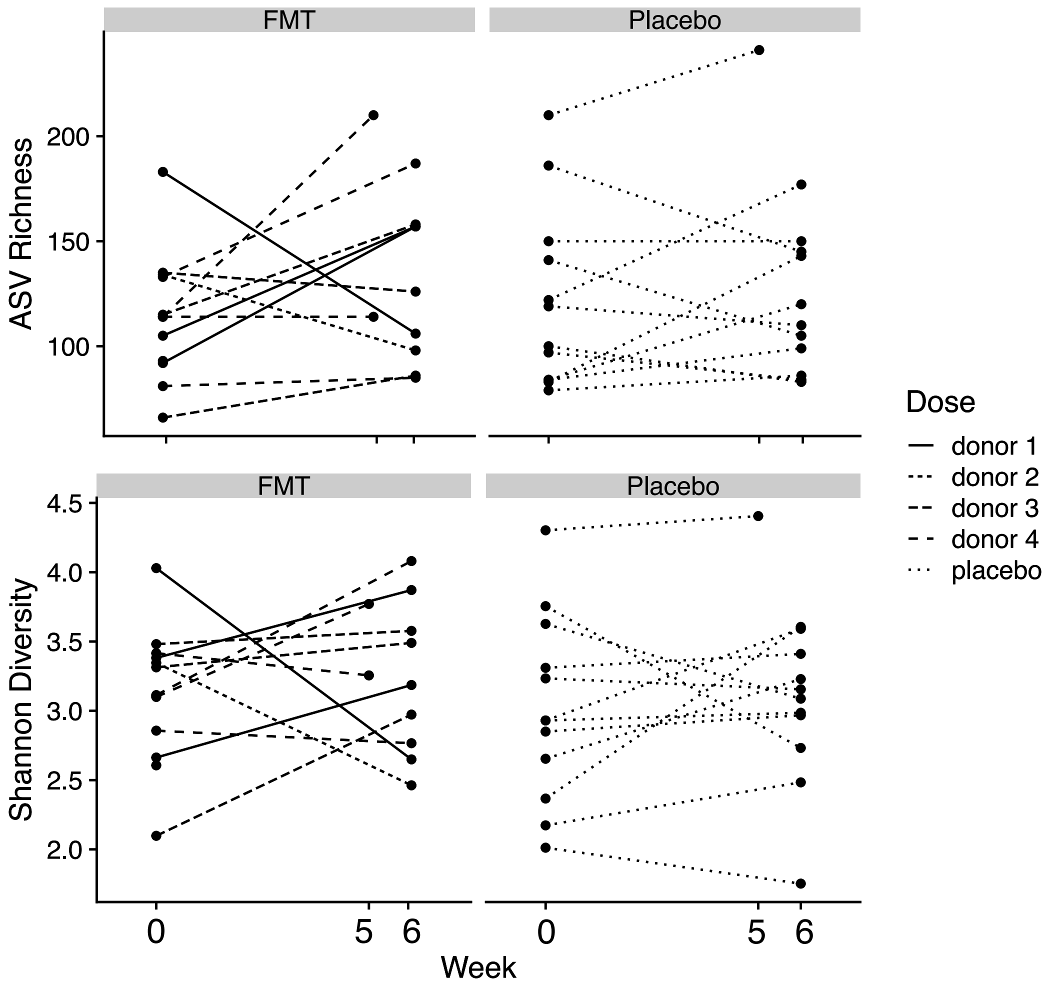

Supplementary Figure J. Beta diversity boxplots displaying microbial species similarity of subject samples to their respective baseline (a) or donor samples (b) as characterized by whole metagenomic sequencing. Plots display data from the three donor 1 recipients and three placebo subjects characterized by shotgun metagenomic sequencing. Placebo results shown in Panel b reflect comparisons between all combinations of placebo subjects to all donor prep samples. Note that one placebo subject was missing a week 1 sample and two placebo subjects were missing a week 12 sample.

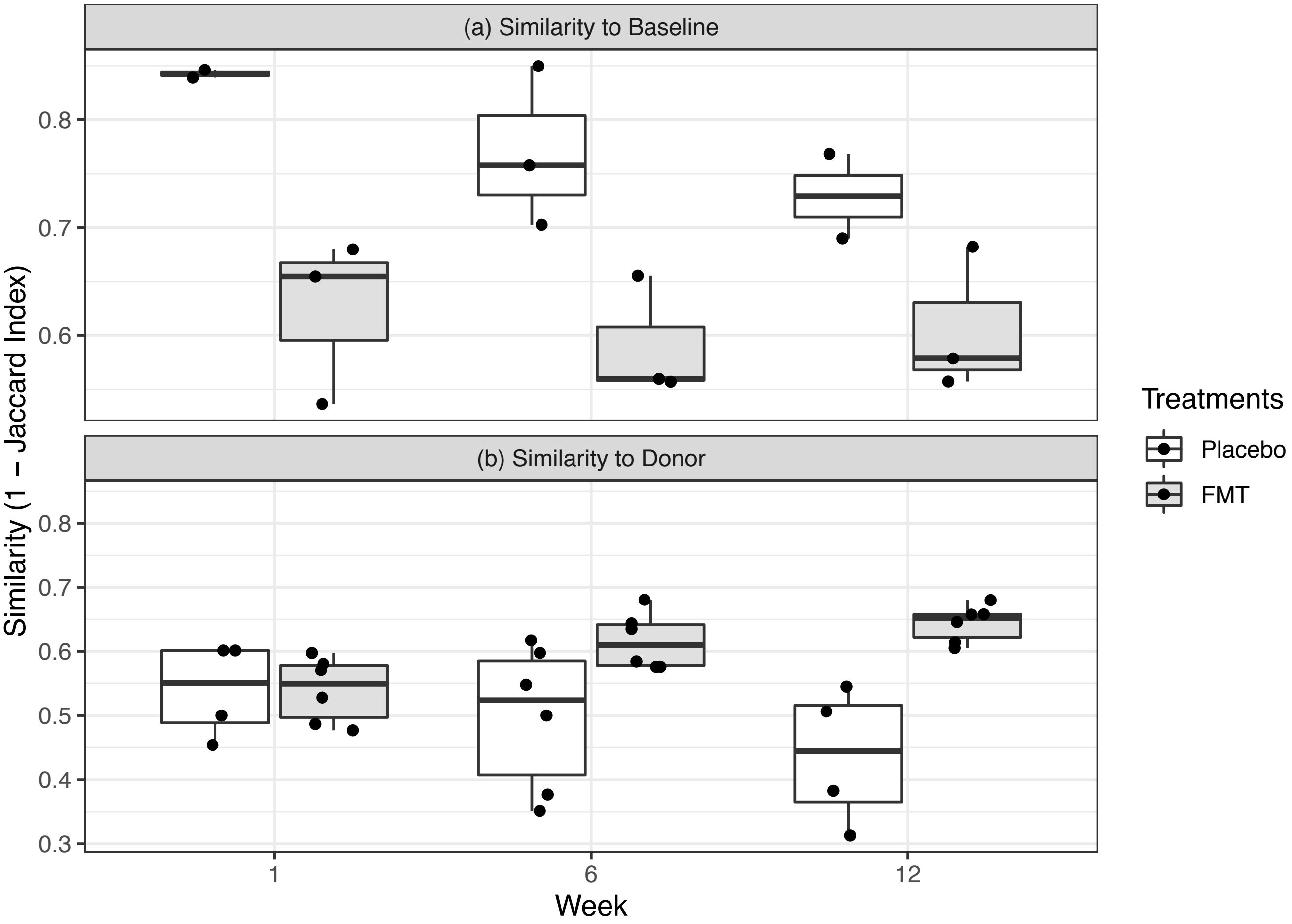

Supplementary Figure K. Phylogenetic distance between conspecific post-FMT subject strains and donor strains. Ratio of the phylogenetic distance between bacterial strains identified in week 1, 6 or 12 samples from three Donor 1 or placebo recipients and either the bacterial strain found in the donor (numerator) or subject baseline (denominator) sample. This analysis was carried out for 99 bacterial species with a representative strain identified in a subject's donor, baseline and post-FMT sample. Values above 1 suggest that the bacterial strain observed in the subject after dosing is more closely related to the subject's baseline strain. Values below 1 suggest the bacterial strain observed in the subject after dosing is more closely related to the donor strain.

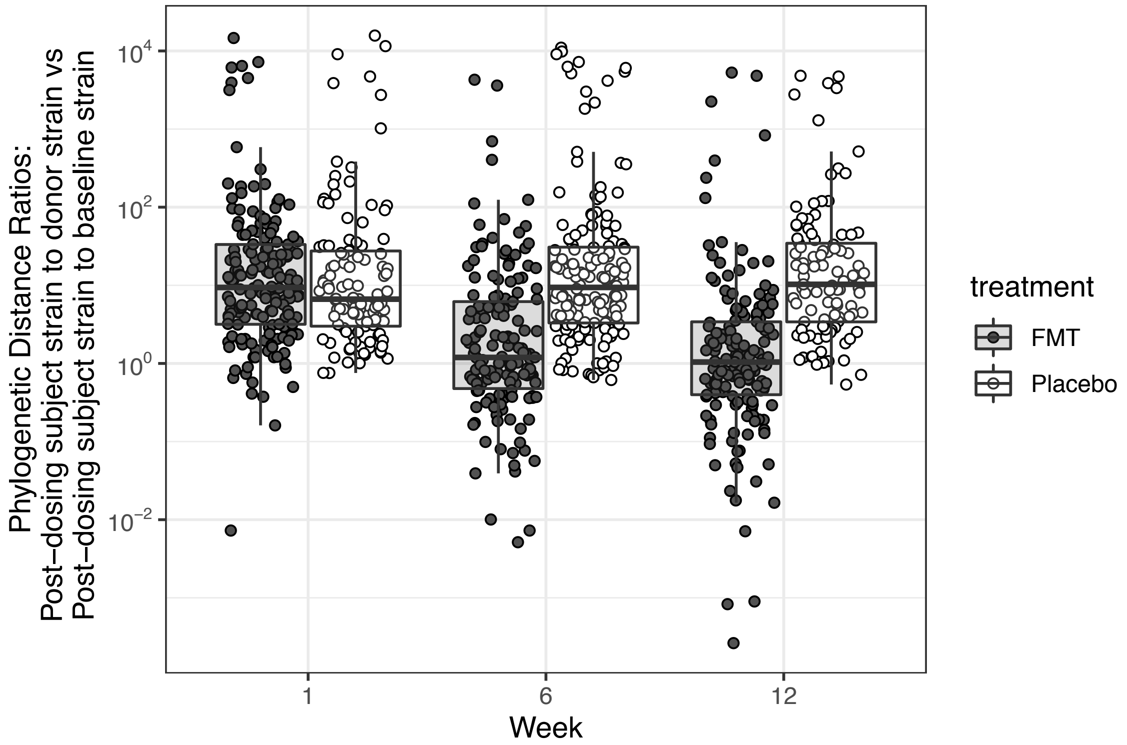

Supplement: S1 Data — (PDF) [file pmed.1003051.s002.pdf]
