# Supplementary material for: Fecal microbiota transplantation for the improvement of metabolism in obesity: The FMT-TRIM double-blind placebo-controlled pilot trial
Source: PLoS Med. 2020 Mar 9;17(3):e1003051. doi: 10.1371/journal.pmed.1003051 (PMC7062239; doi:10.1371/journal.pmed.1003051)
Supplement: S1 Methods — (DOCX) [file pmed.1003051.s003.docx]

SUPPLEMENTARY METHODS

Donor screening

Metabolically healthy donors were selected using criteria adapted from our previously described protocol.^1^ Briefly, inclusion criteria were age 18-50 years old with BMI between 18.5-23 kg/m^2^, no history of BMI >25, and stable weight for the preceding 6 months. Candidates were excluded for any significant past medical history, international travel, or use of antibiotics in the previous 6 months. Eligible donors passed the American Association of Blood Banks donor questionnaire, underwent physical examination and general laboratory screening tests, and passed a rigorous infectious disease screening protocol. In addition, donors were required to have hemoglobin A1c <6.0%, normal lipid panel, and a normal 2-hour oral glucose tolerance test (OGTT).

Preparation of fecal microbiota transplant (FMT) and placebo capsules

FMT capsules were prepared as previously described.^1^ Each donor provided multiple fecal samples and capsules were prepared from samples originating from a single donor (i.e. no mixing of donors). Two to three frozen fecal samples from an individual donor were thawed and blenderized (ambient air), sieved, pelleted and then resuspend to generate frozen capsules. As noted in our published series^1^, 15 capsules contain microbes derived from approximately 24 grams of fecal material, dependent upon the donor diet. Placebo capsules were created using a combination of powdered cocoa and gelatin mixed with a solution of 40% (v/v) glycerol and normal saline (i.e. the same vehicle as the FMT capsules). Both FMT and placebo capsules were doubly encapsulated in size 00 and 0 acid-resistant hypromellose capsules (DRCaps, Capsugel Cambridge, MA, USA), and stored at -80°C. The capsules have no taste, are odorless, and are stored in pharmaceutical vials labelled “study capsules.” The frozen FMT capsules and placebo capsules are identical in terms of visual appearance, weight, and vials. The study was conducted under Investigational New Drug (IND 16643; PI Hohmann) Application to the U.S. Food and Drug Administration.

Rationale for capsule dosing regimen

The dosing regimen of 30 total capsules in the first week was based upon our clinical experience treating patients for recurrent C. difficile colitis with 30 total capsules (15 capsules on each of 2 successive days; described previously).^1^ Additional weekly administrations of 15 capsules were intended to maintain microbiota engraftment and were administered once/week for the convenience of working adults.

Randomization

The 24 study participants were block randomized into FMT and placebo treatment groups in blocks of size 4. There were 6 possible combinations of blocks that distributed treatment equally, and each block was assigned a random order number, which was generated using the RANDBETWEEN function in Excel. The randomization and tracking was done by a single staff member who was not otherwise interacting with participants. All other study staff and participants were blinded until 12 weeks.

Hyperinsulinemic euglycemic clamps

We assessed peripheral insulin sensitivity at 0 and 6 weeks with hyperinsulinemic euglycemic clamps. Following a 12 hour fast, intravenous infusions of insulin and dextrose 20% (D20) were administered. A priming dose of insulin was infused at a rate of 300 mU/m^2^/min for the first 2 minutes, followed by a continuous infusion of 60 mU/m^2^/min for the next 118 minutes. A variable D20 infusion rate was adjusted to maintain a target blood glucose level of 90 mg/dL. Arterialized blood glucose was evaluated every 5 minutes using a B-Glucose Analyzer (Hemocue, Lake Forest, CA). Blood samples for later analysis were collected prior to the start of the insulin infusion, and at the 80, 100, and 120 minute timepoints. The method of DeFronzo et al.^2^ was adapted to calculate the insulin stimulated glucose uptake (M) for the interval between 100-120 minutes, with M adjusted for lean body mass. Additional analyses were performed with M normalized for steady state insulin level (M/I).

Fecal sample collection

Donor stool samples were obtained as described in Methods above, and triplicate samples of each batch of FMT capsule preparations were sequenced. Stool samples were obtained from study participants at 0, 1, 3, 5, 6, and 12 weeks. Study participants produced samples within 12 hours of study visits and stored samples in an insulated transport container with frozen gel packs until delivered to the study staff. On receipt of the samples, approximately 0.5 mL aliquots were made either undiluted, or vortexed with 1 mL of either 95% ethanol or 15% glycerol in sterile water. Aliquots were then stored in a -80 C freezer until analyzed.

Three participants only submitted stool samples for microbiome analysis at 5 of the 6 study timepoints. Four microbiome samples were removed prior to analysis due to low DNA sequencing depth. This yielded microbiome data for 133 subject timepoints and triplicate subsamples of 9 donor stool prep samples.

Microbiome sequencing

Donor and study participant microbiomes were characterized by 16S amplicon sequencing. Triplicate aliquots from each donor prep, and single samples from each participant timepoint were individually processed. Genomic DNA was extracted using the PowerMag Soil DNA Isolation Kit (MOBIO Laboratories, Catalog No. 27100) following the manufacturer’s instructions. 16S rDNA V4 sequences were PCR-amplified from 1μl of DNA extract using the AccuPrime High Fidelity PCR kit (Invitrogen Catalog No 12346094) with the primer pair 515F (5’ AGCMGCCGCGGTAA 3’) and 806R (5’ GGACTACHVGGGTWTCTAAT ‘3) containing Illumina MiSeq adaptors and single-end barcodes. The following PCR temperature cycles were used: 98°C for 3 seconds, 33 cycles of: 98°C for 20 seconds, 50°C for 30 seconds, 72°C for 90 seconds; then 72°C for a final 10 minutes. PCR yields were quantified using Invitrogen’s Quant-iT Picogreen dsDNA assay kit (Catalog No P7589). Amplicons were then pooled in equal quantities, cleaned with AMPure beads (Beckman Coulter) and paired-end sequenced on the MiSeq platform following Nextera XT library preparation (Illumina).

16S V4 Data Processing and Diversity Analyses

16S V4 sequencing reads were demultiplexed using the split_libraries_fastq.py function in QIIME (QIIME version 1.9.1)^3^ and sequences were quality trimmed using the DADA2 pipeline (dada2 version 1.4.0)^4^ in R (version 3.3.2) with the parameters: EE=2, TruncL= c(200, 180) and q=10. The set of unique 16S V4 DNA sequences, referred to as amplicon sequence variants (ASV), were then inferred using Dada2 and an ASV table of read counts per ASV per sample was generated. ASVs were taxonomically classified using the RDP classifier^5^ with the SILVA 16S rRNA database (Silva nr v128).^6^ A neighbor-joining phylogenetic tree was built with the phangorn R package (version 2.4).^7^ For alpha and beta diversity analyses, the ASV table was down-sampled to 17,000 reads per sample using the rrarefy command in the vegan package to standardize sequencing depth.^8^ The PCoA was generating using the Ampvis2 R package (Ampvis2 version 2.4.4).^9^

In order to capture the replacement or addition of bacteria taxa following FMT we calculated Jaccard distances between 1) post-dosing participant time points and their paired baseline samples and 2) each participant time point to their corresponding donor material.^10^ For our purpose the Jaccard Index is more appropriate than the popular Unifrac metric because we are only interested in the presence or absence of specific taxa not changes in phylogenetic distances between organisms across samples. Plotted similarity values for donor-to-placebo participant comparisons reflect all pairwise combinations of placebo participants and triplicate donor prep samples. We hypothesized that FMTs would result in participant microbiomes that were more similar to paired donor samples and less similar to baseline samples compared to placebo, therefore we used one-sided Wilcox rank sum tests. The multiple similarity values corresponding to each participant-donor-pairing, as a result of triplicate donor samples and preps, were averaged prior to Wilcox tests resulting in 12 placebo and 12 FMT values per time point.

Protocol amendments

The original study protocol was approved by the Partners Human Research Committee on 3/24/2016. Prior to the first study participant enrollment (8/25/16), several study design elements were adjusted (e.g. size and length of study, eligibility criteria, outcome measures) as additional expertise was added and sources of funding were secured. The initial primary outcome of body weight was changed to insulin sensitivity on 9/12/16 after discussion among the Principal Investigators and in recognition that prior FMT studies had only observed changes in insulin sensitivity, and not body weight. This change occurred <3 weeks after the enrollment of the first study participant but before any follow-up outcome measures had been obtained. No other major protocol changes occurred after this date.

**REFERENCES**

1. Youngster I, Russell GH, Pindar C, Ziv-Baran T, Sauk J, Hohmann EL. Oral, capsulized, frozen fecal microbiota transplantation for relapsing Clostridium difficile infection. *JAMA* 2014; **312**(17): 1772-8.

2. DeFronzo RA, Tobin JD, Andres R. Glucose clamp technique: a method for quantifying insulin secretion and resistance. *Am J Physiol* 1979; **237**(3): E214-23.

3. Caporaso JG, Kuczynski J, Stombaugh J, et al. QIIME allows analysis of high-throughput community sequencing data. *Nat Methods* 2010; **7**(5): 335-6.

4. Callahan BJ, McMurdie PJ, Rosen MJ, Han AW, Johnson AJ, Holmes SP. DADA2: High-resolution sample inference from Illumina amplicon data. *Nat Methods* 2016; **13**(7): 581-3.

5. Wang Q, Garrity GM, Tiedje JM, Cole JR. Naive Bayesian classifier for rapid assignment of rRNA sequences into the new bacterial taxonomy. *Applied and environmental microbiology* 2007; **73**(16): 5261-7.

6. Quast C, Pruesse E, Yilmaz P, et al. The SILVA ribosomal RNA gene database project: improved data processing and web-based tools. *Nucleic Acids Res* 2013; **41**(Database issue): D590-6.

7. Schliep KP. phangorn: phylogenetic analysis in R. *Bioinformatics* 2011; **27**(4): 592-3.

8. Oksanen J, Blanchet FG, Friendly M, et al. Package ‘vegan’. 2019. <https://cran.r-project.org/web/packages/vegan/index.html>.

9. Skytte Andersen KS, Kirkegaard RH, Karst SM, Albertsen M. ampvis2: an R package to analyse and visualise 16S rRNA amplicon data. *bioRxiv* 2018: doi:10.1101/299537.

10. Lozupone C, Knight R. UniFrac: a new phylogenetic method for comparing microbial communities. *Applied and environmental microbiology* 2005; **71**(12): 8228-35.
