# Supplementary material for: Fecal microbiota transplantation for the improvement of metabolism in obesity: The FMT-TRIM double-blind placebo-controlled pilot trial
Source: PLoS Med. 2020 Mar 9;17(3):e1003051. doi: 10.1371/journal.pmed.1003051 (PMC7062239; doi:10.1371/journal.pmed.1003051)
Supplement: S1 Obesity FMT Protocol — (DOCX) [file pmed.1003051.s004.docx]

**Placebo Controlled Study of Fecal Microbiota Transplant (FMT) to Impact Body Weight and Glycemic Control in Adults using a Frozen Encapsulated Inoculum**

**Massachusetts General Hospital**

**Endocrine Unit and Infectious Diseases Division**

**PI: Elaine W. Yu, MD**

**Co-I: Elizabeth L. Hohmann, MD**

**Study Coordinators: Petr Stastka, Mariam Torres**

**Protocol Version 5.6**

**Date: March 6, 2019**

**IND Number: 16643**

**PHRC Protocol Number: 2015P001632/MGH**

**CT.gov number: NCT02530385**

**I. Background and Significance**

Multiple lines of evidence suggest that gut microbiota play an important role in regulating human metabolism via effects on body weight and systemic insulin resistance.[^1^](#_ENREF_1) In rodents, manipulation of the gut flora with fecal microbiota transfer (FMT) induces weight loss and prevents development of obesity.[^2^](#_ENREF_2)^,^[^3^](#_ENREF_3) In human studies, FMT administered via duodenal bowel lavage may confer benefits of short-term improvements in metabolic endpoints including insulin sensitivity.[^4^](#_ENREF_4) Recently, a new protocol for FMT has been developed at MGH utilizing oral capsulization that greatly improves the safety and feasibility of FMT as an investigative and therapeutic tool.[^5^](#_ENREF_5)

Few safe and effective treatments for obesity

Over one-third of U.S. adults are obese[^6^](#_ENREF_6) and nearly a quarter have metabolic syndrome^[7](#_ENREF_7)^. Obesity and the metabolic syndrome are associated with disabling comorbidities and an increased risk for cardiovascular disease and mortality.[^8^](#_ENREF_8) Safe and effective treatments for obesity have been difficult to discover; indeed 4 FDA-approved drugs have been withdrawn from the market due to safety concerns. Bariatric surgery, while effective, is accompanied by high costs and potential morbidities and is therefore reserved for the most severe categories of obesity. Finding noninvasive low-cost alternatives to improve obesity and human metabolism is an imperative for our society.

Effects of gut microbiota on energy balance

The gut microbiota is a diverse collection of several thousand species that actively participate in gut physiology and metabolic energy balance. These microbial species have been postulated to modulate energy absorption, gut motility, appetite, glucose and lipid metabolism, and hepatic fatty storage.[^9^](#_ENREF_9) The precise mechanisms by which gut microbiota perform these tasks are unclear. The dominant theories involve (1) increased energy harvesting from fermentation of foods otherwise indigestible to the host, (2) enlarging the nutrient absorption surface by promoting the intestinal epithelial growth, and (3) interaction with enteroendocrine cells to promote hormonal changes that influence host appetite and satiety, and (4) absorption of bacterial fragments such as lipopolysaccharides leading to “metabolic endotoxinemia”, which promotes obesity and hyperinsulinemia.

Gut microbiome and obesity

Intestinal bacteria have been implicated in the etiology of obesity.[^1^](#_ENREF_1) Animal studies have demonstrated that germ-free mice have lower body fat content than conventionally raised mice, an effect that is independent of diet.[^10^](#_ENREF_10) These germ-free mice are also resistant to diet-induced obesity.[^11^](#_ENREF_11) Genetically obese mice (ob/ob) have a reduction in the relative abundance of Bacteroidetes, with a proportional increase in Firmicutes.[^12^](#_ENREF_12) Importantly, similarly altered ratios of firmicutes:bacteroidetes and overall reductions in microbial diversity have been demonstrated in comparisons of obese and lean adults[^12-14^](#_ENREF_12) and in diabetic adults[^15^](#_ENREF_15), although this finding has not been verified in all studies.[^16^](#_ENREF_16)^,^[^17^](#_ENREF_17) Other studies have found that the obese state is accompanied by overall reductions in microbial diversity and a relative increase in pathogenic bacteria.[^18^](#_ENREF_18)^,^[^19^](#_ENREF_19)

Metabolic effects of altering the gut microbiome

Antibiotics are known to alter the community structure of the gut microbiome, and are associated with increased risk of becoming overweight in both animal[^20^](#_ENREF_20)^,^[^21^](#_ENREF_21) and human[^22^](#_ENREF_22)^,^[^23^](#_ENREF_23) studies. Interestingly, 7-day treatment with oral vancomycin led to a decrease in microbial diversity and was associated with a decrease in insulin sensitivity among men with metabolic syndrome.[^24^](#_ENREF_24)

A more directed method of altering the microbiome involves the direct delivery of donor fecal material into the gut. Colonization of germ-free mice with intestinal flora from obese mice led to greater increases in body weight and fat tissue than colonization with flora from lean mice.[^25^](#_ENREF_25) Other studies demonstrate that fecal microbiota transfer (FMT) from thin or obese adults can transmit the lean or fat phenotype when transplanted into mice, in the setting of the correct dietary conditions.[^3^](#_ENREF_3) Furthermore, fecal transplants from mice who sustained weight loss after gastric bypass surgery led to weight loss and improvements in insulin sensitivity among germ-free mice recipients.[^2^](#_ENREF_2) In human studies, fecal transfer from lean donors delivered at a single timepoint by duodenal bowel lavage led to improvements in insulin sensitivity at 6 weeks among men with metabolic syndrome, even in the absence of a dietary intervention.[^4^](#_ENREF_4)

Skeletal effects of altering the gut microbiome

Recent data suggest that the gut microbiome may also have a direct impact on bone health. Germ-free mice have increased bone mass and decreased osteoclast number, and transplant of gut microbiota into germ-free mice led to normalization of bone mass.^26^ Antibiotics and probiotics causing shifts in the microbiota spectra can also increase bone mass in animals models (Ohlsson et al Endo Metab 2014).^27^ Mouse models have indicated that the gut microbiome has an effect on the immune system, hormonal pathways, and production of bacterial metabolites that could signal bone cells and affect bone density.^28^

Oral capsulized FMT protocol

Standard FMT protocols involve invasive procedures such as colonoscopy, nasogastric / duodenal tubes, or retention enemas, which are accompanied by procedural challenges, risks, and costs. Recently, we have developed a new FMT protocol that involves the capsulization of frozen fecal material.[^5^](#_ENREF_5) These oral capsules were highly effective in resolving otherwise refractory cases of *C. difficile* infection. The increased ease, tolerability, and safety of oral administration now allow study of FMT for sustained therapeutic intervention. This extended exposure is a major advance, and more likely to durably alter the microbiome.

Contributions of this study to the field

We believe that transfer of the “lean” microbiota to an obese recipient may lead to weight loss and metabolic improvements. Therefore, we propose to conduct a randomized double-blind placebo-controlled pilot study to evaluate the safety and effectiveness of oral FMT administration on weight and metabolism in obese adults. This highly innovative study will leverage this new non-invasive FMT technique to promote durable changes in the gut microbiome, and could represent a new horizon of treatments for obesity and insulin resistance. Future studies including microbiomic analysis may identify specific strains of beneficial organisms to help narrow treatments from generic FMT to specific probiotic strains. Identification of specific microbes and metagenomic pathways will also aid in our understanding of the mechanism by which the gut microbiome controls response metabolism.

**II. Specific Aims**

We propose a randomized placebeo-controlled trial to study the impact of gut microbiome alterations on weight, insulin sensitivity, and other important metabolic endpoints.

**Aim 1**. To identify and characterize healthy lean donors from a phenotypic perspective to generate FMT capsules for a clinical trial in obese subjects.

**Aim 2.** To determine the impact of oral capsulized FMT from healthy lean donors on insulin sensitivity in obese adults in a 3-month randomized double-blind placebo-controlled trial. Secondary outcomes include changes in body weight, metabolic markers, and FMT-related adverse events. We hypothesize that FMT recipients will experience an improvement in insulin sensitivity and other metabolic endpoints. An optional extension study will evaluate the durability of gut microbiome change at 12 months.

**Aim 3.** To explore associations between the gut microbiome and bone density.

**III. PATIENT/SUBJECT SELECTION**

Lean donors for FMT are being recruited from Dr. Hohmann’s IRB-approved protocol 2014P002706 “Placebo Controlled Study of Fecal Microbiota Transplant (FMT) for a second episode of *C. difficile* infection in adults using a frozen encapsulated inoculum”. Lean donors must meet all the eligibility criteria as noted in protocol 2014P002706. Briefly, they must be age 20-60 years, have no significant past medical history, no use of antibiotics for the previous 6 months, and must pass a rigorous infectious disease screening protocol. In addition, donors must pass stricter weight and metabolic criteria than as outlined in protocol 2014P002706; in particular, they must have a BMI 18.5-23 kg/m2 with no history of BMI exceeding 25 other than for pregnancy, stable weight for the preceding 6 months, and they must pass an 2-hour oral glucose tolerance test (fasting glucose <100 mg/dl, 2 hour OGTT glucose <140 mg/dl) to be eligible to donate for this obesity study.

24 obese recipients will be recruited to participate in the study from the greater Boston area. The obese recipients will be randomized 1:1 to weekly FMT vs. placebo capsules (n=12 FMT, n=12 placebo) for 6 weeks, with a total follow-up time of 24 weeks, or 12 months if they choose to participate in the optional substudy.

Inclusion criteria

- BMI ≥30 kg/m2 and stable weight for the previous 3 months
- Willingness to accept risk of unrelated donor stool.
- Age 25-60
- Able to consent for self in English language
- Subjects are not expected to receive antibiotics in the next 12 weeks.
- Patient must be willing to have screening labs for Hepatitis B and C, and HIV (as required by the FDA for past studies).
- Mild-moderate abnormal glucose metabolism: HOMA-IR >2.0 or fasting plasma glucose ≥ 100 mg/dl; however subjects will be excluded for HOMA-IR >8.0

Exclusion criteria

- Initiation or discontinuation of a medication known to affect body weight or insulin sensitivity within 3 months of enrollment (e.g. hormonal contraception, psychiatric medications: tricyclic antidepressants, monoamine oxidase inhibitors, paroxetine, escitalopram, lithium, olanzapine, clozapin, risperidone, carbamazepine, valproate, divalproex, mirtazapine)
- Use of weight-loss medications or diabetic medications in the preceding 1 year (e.g. metformin, sulfonylureas, insulin, etc)
- Antibiotic use in the preceding 6 months
- Delayed gastric emptying syndrome
- Known chronic aspiration
- Swallowing dysfunction or oral-motor dyscoordination, or inability or unwillingness to swallow multiple large capsules
- Malabsorptive disorders including celiac disease, cystic fibrosis, irritable bowel syndrome, prior gastrectomy, bariatric surgery and any state of chronic diarrhea
- Pregnant women or women trying to conceive or women who are breastfeeding; women of child-bearing potential will have a urine or serum hCG test.
- Patients with an acute illness or an acute exacerbation of underlying co-morbid condition
- Subjects on high dose steroids (>40 mg daily)
- Subjects on multiple/combination immunosuppressive regimens including high dose corticosteroids, calcineurin inhibitors, mTOR inhibitors, lymphocyte depleting biologic agents, anti-TNF agents.
- Patients with a history of HIV, hepatitis B, hepatitis C, decompensated cirrhosis, recent bone marrow transplant, other causes of severe immunodeficiency.
- Patients with a history of significant allergy to foods not excluded from the donor diet (excluded foods are tree nuts, peanuts, shellfish, eggs)
- Allergy to chocolate/cocoa or gelatin, or unwillingness to ingest gelatin (in placebo capsules).
- Screening labs: AST or ALT >3x normal; Hematocrit <33%; White Blood Cell count <3000 cells/uL; ANC<500; Creatinine >2.0 mg/dL
- Patients with abnormal bowel habits, e.g. constipation with <4 bowel movements/week

**IV. SUBJECT ENROLLMENT**

Methods of enrollment:

Obese adults will be recruited from the MGH Weight Center. The PI does not perform clinical work at the MGH Weight Center. However, if a patient of the PI’s is identified who may be eligible for the study, the PI will ask a physician colleague to explain the study and will provide patients the Consent Form to review at home. In addition, recruiting advertisements will be posted on email, the internet, and by flyer. Finally, we will add the study to the https://register.clinicaltrials.gov database. All letters and advertisements will be approved by the Partners IRB before they are used. All eligible obese subjects will be invited to participate in the hyperinsulinemic-euglycemic clamp substudy.

Optional Substudies

All participants will be asked if they are interested in participating in the optional substudies as part of the initial screening process. The first substudy is to examine associations of gut microbiome with bone density (Aim 3). The second substudy is an extension the main study’s stool and blood sampling to determine the longevity of any effects observed as a result of FMT. If participants express interest, they will be enrolled in the substudies.

Informed consent

Written informed consent will be obtained by a nurse practitioner or physician investigator. In situations where a nurse practitioner is obtaining consent, subjects will be offered the option for further discussion with a physician investigator if they have any remaining questions. This offer will be documented in the research record.

Any consent problems will be reported to the PHRC in real-time.

Randomization

Obese recipients will be randomized to receive either FMT or placebo capsules in a 1:1 ratio. Double blinding is important. Subjects will be assigned by a computer generated randomization order known to an individual not involved in assessing the patients. This individual will identify the assignment, take appropriate capsules from relevant freezer locations, and place them on dry ice as usual for administration, but place them in a vial labelled “STUDY Caps” for administration so neither physician nor subject will be able to identify real vs placebo capsules. The actual labeled containers identifying caps as real or placebo will then be sealed in an opaque envelope and saved for review at the time of unblinding. This will serve as a “double check” on actual assignment.

**V. Study procedures**

Lean donors will undergo testing and follow procedures outlined in protocol 2014P002706. Main procedures will occur in the primary 12-week study period; a supplemental week 24 phone-call will be performed for a safety check. In addition, samples of the fecal collection will be stored at -80°C for further microbiome analysis.

Obese recipients will undergo the following procedures:

Phone Screen (10-20 minutes)

Screen Visit (30 minutes)

- Review eligibility and understand/see size of capsules
- Obtain informed consent
- Height, weight measurements to determine eligibility
- Screening labs: Complete blood cell count w/ differential, comprehensive metabolic panel, HIV Ab, HBV sAb/sAg, HCV Ab, fasting insulin and glucose.
- Subjects will be counseled not to start any new major dietary changes, exercise regimens, or medications throughout the primary 12-week study period
- Subjects will be given information about how to perform fecal collections (see Stool Collection document)

Baseline Visit, Week 0 (Day 1: 5 hours, Day 2: 15 minutes, Day 3: 15 minutes)

- Fasting blood samples for measurements of HOMA-IR, lipids, CRP, and HbA1c.
- Women of child bearing potential will have a urine pregnancy test
- Height, weight measurements
- 48-hour food recall
- Whole body DXA scan for body composition analysis
- DXA scan of lumbar spine and hip to assess baseline BMD (optional substudy)
- Fecal collection
- Indirect Calorimetry
- Hyperinsulinemic euglycemic clamp
- After above study procedures are complete, subjects will receive FMT (or placebo) caps, 15 capsules on each of 2 successive days. Note that participants will need to either fast for 4 hours after the clamp or return on the subsequent day for their first capsule administration.

Weeks 1-5 (15 minutes)

- Interval side effect assessment (see Side Effect Assessment document)
- Subjects return for weekly administration of 15 capsules (for a total of 6 weeks, including Week 0)
- Fecal collection at weeks 1, 3 and 5.

Week 6 (5 hours)

- Interval side effect assessment (see Side Effect Assessment document)
- Height, weight measurements
- Women of child bearing potential will have a urine pregnancy test
- Fasting blood samples for measurements of HOMA-IR, lipids, CRP, and HbA1c.
- 48-hour food recall
- Fecal collection
- Whole body DXA scan for body composition analysis
- Fecal collection
- Indirect Calorimetry
- Hyperinsulinemic euglycemic clamp
- Note that we will allow a +1 week window for scheduling the week 6 visit

Week 12 (1 hour)

- Interval side effect assessment (see Side Effect Assessment document)
- Height, weight measurements
- Fasting blood samples for measurements of HOMA-IR, lipids, CRP and HbA1c.
- 48-hour food recall
- Women of child bearing potential will have a urine pregnancy test
- Whole body DXA scan for body composition analysis
- Fecal Collection

Week 24 (Month 6) Follow Up (5-20 minutes)

- If not enrolled in the extension study, interval side effect and self-reported weight assessment will occur by phone (see Phone Follow-Up document)
- If enrolled in the optional extension substudy, subjects will come for an in-person visit to MGH.
- Fecal collection
- Fasting blood samples for measurements of HOMA-IR, lipids, CRP and HbA1c.
- Interval side effect assessment will be conducted.
- Note that we will allow a ±2 month window for scheduling this visit

Month 12 (1 hour; optional extension study)

- Interval side effect assessment (see Side Effect Assessment document)
- Height, weight measurements
- Fasting blood samples for measurements of HOMA-IR, lipids, CRP and HbA1c.
- 48-hour food recall
- Women of child bearing potential will have a urine pregnancy test
- Whole body DXA scan for body composition analysis and DXA scan of lumbar spine and proximal femur to assess bone mineral density
- Fecal Collection
- Note that we will allow a ±2 month window for scheduling this visit

**C. Technical Methods**

Phone screen: Phone discussion to explain approach and alternatives briefly (10-20 minutes). The purpose of this discussion is to ensure subjects who cannot get past the aesthetic concerns of this procedure do not spend time coming to the hospital for an in person discussion. People may wish time to consider after having this discussion. Those who wish to have an in person discussion, even if not decided upon having the procedure are welcome to come in for further discussion. The consent form is provided at this time, if desired.

FMT and placebo capsule preparation: We will generate FMT capsules from donors per our FDA-approved operating procedures. Donors will be asked to refrain from eating common allergens within 5 days of the donation (tree nuts, eggs, peanuts, shellfish) but otherwise not alter their diets. Volunteers will have an interim health query for febrile, system, and GI symptoms at the time of donation, and defer collection if there is any change in health status. Processing is carried out under aerobic conditions. A fecal suspension is generated in normal saline without preservatives. Materials are sequentially sieved to remove particulate material. The final slurry is concentrated by centrifugation and re-suspended in saline at 1/10 volume of the initial sample with 10% glycerol added as a bacterial cryoprotectant. Fecal matter solution is pipetted in size 0 capsules (650µL), which are closed and then secondarily sealed in size 00 capsules. Capsules are stored frozen at -80^0^C for up to 6 months at which point they are discarded if not used. 1-2 hours prior to administration they will be transferred to -20^0^C and then transported to the dosing site on dry ice. Commercially available acid-resistant hypromellose capsules (DRCaps, Capsugel, Cambridge, MA, USA) are used. Each inoculum is prepared from the feces of a single donor. This project is under FDA IND #16643.


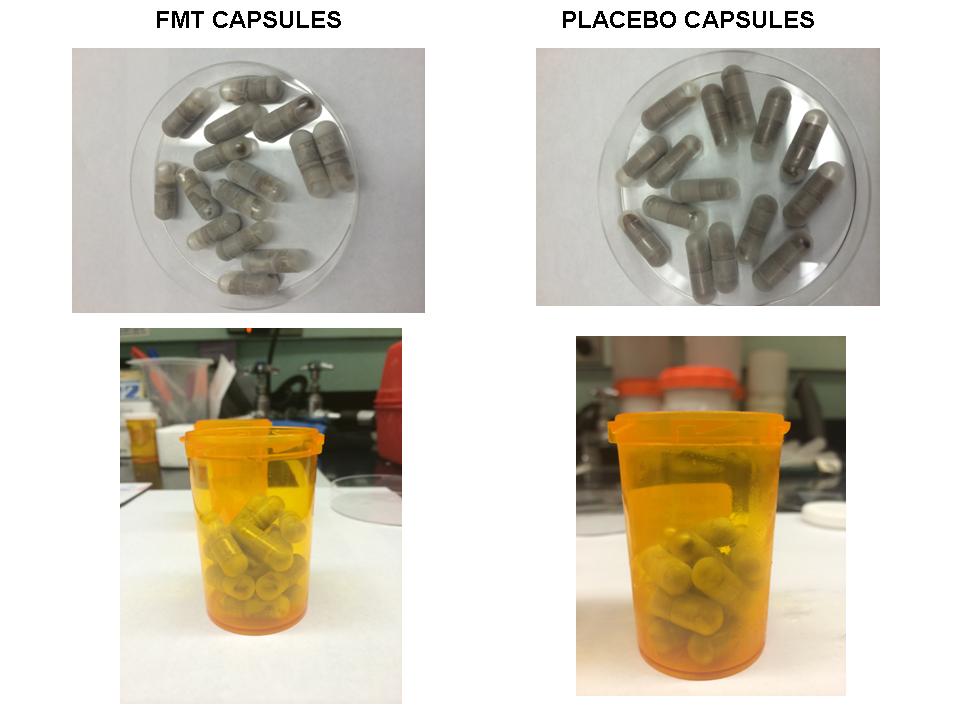
 Photo credit: Mariam Torres Soto

Placebo capsules will consist of a combination of powdered cocoa and gelatin in normal saline/glycerol (same vehicle as FMT capsules). Gelatin is added to make the placebo solution more viscous and physically fill capsules in the same way that the standard FMT inoculum does for appearance purposes. Placebo capsules are identical in appearance to FMT capsules, and are included in the above mentioned FDA IND # 16643.

FMT and placebo capsule administration: Subjects will be NPO at least 4 h prior to and for 1 hour following capsule intake on each day, to minimize risk of vomiting during ingestion of capsules. Subjects will be handed each capsule by an investigator and asked to take each capsule with a sip of water. Subjects will be asked to drink at least 12 oz of water during administration to facilitate dilution of stomach contents and transit into the small intestine. In the event of vomiting subjects will be observed, and not re-dosed.

Subjects are evaluated after taking capsules by the study investigators, typically for 15 minutes. Immediate side effects are very uncommon in our experience. Capsules are odorless and tasteless and do not dissolve in the acidic environment of the stomach. (In our study of capsules to date we have had 1/61 subjects who complained of nausea during administration, and this individual had concurrent psychiatric illness and concurrent irritable bowel syndrome diagnosed by an outside gastroenterologist. One child vomited 4 hours after capsules were administered.).

Subjects receive a copy of the signed consent form to take home and an information sheet (attached) on symptoms to monitor and study physician phone numbers. All patients have our contact numbers and will be encouraged to call for any concerns. Subjects are asked to inform us if they require the initiation of antibiotics for intercurrent clinical illness, during the study period.

Subjects will receive capsules at weekly intervals (± 2 days) for weeks 0-5.

Subject counseling: Subjects will be counseled to remain on the same diet that they are currently on and not to initiate any major exercise regimens throughout the primary 12 week study period. In addition, women with child-bearing potential will be informed that they need to use contraception throughout the study. If they are taking a hormonal contraceptive, they need to remain on such until the end of the study. If they are not using a hormonal contraceptive, they will need to use other forms of birth control, such as barrier methods with a spermicide, IUD, or abstinence. We will ask that they not actively initiate or discontinue hormonal treatments during the study, as this may confound our assessments of body weight and insulin sensitivity.

Height, weight measurements: Height and weight measurements will be performed in triplicate in light clothing without shoes. Waist circumference will be measured as the narrowest circumference between the lower costal margin and iliac crest in the standing position at the end of a normal exhalation. All measurements will be performed using standardized protocols by bionutritionists at the MGH Clinical Research Center.

Fecal sampling: Fecal samples will be collected from recipients at weeks 0, 1, 3, 5, 6, and 12 (and 6 and 12 months if subjects choose to participate in the optional extension study), and stored in a -80°C freezer according to established protocols.[^2^](#_ENREF_26)^8^ Recipients will be given instructions and stool collection kits to bring home (see Stool Collection document). All samples will be brought to MGH by the patient.

DXA: Whole body, hip and spine DXA scans will be performed. DXA scans of the whole body will be obtained at baseline, 6 and 12 weeks (and 12 months if subjects choose to participate in the optional extension study). For those participating in the optional bone density substudy, DXA scans on the spine and hip will be obtained at baseline and 12 months. Measurements will be performed on QDR Hologic Discovery A machines. Body composition (lean body, fat mass including subcutaneous and visceral compartments) and bone density (PA spine, total hip, and femoral neck) will be measured using standard DXA analysis procedures as recommended by the manufacturer. The effective absorbed radiation dose for each full body DXA scan is 0.0084 mSv and for the hip and spine DXA scans is 0.022 mSv. The total amount of radiation from participation in this study and the optional extension study is 0.076 mSv for these procedures.

Hyperinsulinemic-euglycemic clamp: Subjects will undergo clamp studies to assess peripheral insulin sensitivity at week 0 and week 6. This procedure is performed at the MGH CRC. Peripheral insulin sensitivity will be determined during the last 20 minutes of the clamp using the DeFronzo method.[^29^](#_ENREF_27) Clamp procedure will be as follows.

- Subjects will be asked for fast for 12 hours prior to their study visit.
- Insulin infusate will be prepared by the MGH pharmacy.
- Two intravenous (IV) catheters will be inserted:  (1) one placed in the hand when access is available, otherwise in the forearm or antecubital space, to be used for venous sampling; (2) one placed in the antecubital space or proximal forearm and used for administration of insulin and 20% dextrose as below.
- The hand/forearm with the IV that will be used for venous sampling will be placed in a warming box for the duration of the clamp procedure, although it may be withdrawn if the subject finds the warmth uncomfortable.
- At time “0” (approximately 9am), a priming dose of 300 mU/m^2^/min of insulin will be given for 2 minutes followed by a continuous infusion of 60 mU/m^2^/min for the next 118 minutes.
- From 0-120 minutes, a sample (≤ 0.5cc) of venous blood will be drawn every 5 minutes for assessment of glucose using a Hemocue glucose analyzer.  Each sample is run twice in the Hemocue analyzer, with an additional third run if the difference between the first two runs is ≥4mg/dL.  Samples for serum insulin concentration will be drawn at times 0, 80, 100, 120 minutes.
- Starting at 5 minutes, a variable 20% dextrose infusion will be administered to achieve a target glucose of 90mg/dL (range 85-95mg/dL).  The rate will be adjusted by the investigator, an MD or NP who is trained in clamp procedure.  The initial infusion rate of 20% dextrose will be 0-30cc/hr, with the exact rate determined by the investigator based on the subject’s fasting glucose, available clinical information, and, if available, data from the subject’s previous clamp procedure for the study.  (For example, a subject who is known clinically to be insulin resistant and has a fasting glucose of 110mg/dL would be started at a rate of 0cc/hr, whereas a lean, muscular subject who exercises regularly and has a fasting glucose of 70mg/dL would be started at 30cc/hr.)
- The rate of 20% dextrose infusion will be adjusted up or down for the duration of the clamp procedure by the investigator.  Rate changes of 0-40cc/hr will be made by the investigator every 5 minutes, based on the subject’s venous glucose and rate of change in glucose, to achieve target glucose of 90mg/dL.
- At 120 minutes, the insulin infusion will be discontinued, and the glucose infusion will continue for 30 more minutes.  During this time, subjects will be given a meal.  At 150 minutes, venous glucose will be checked, and the glucose infusion will be discontinued if venous glucose ≥80mg/dL.  If glucose is <80mg/dL, the glucose infusion will be continued and will be weaned by the investigator at the bedside, with repeat venous glucose sampling every 10 minutes until the glucose is ≥80mg/dL and the infusion is stopped.
- After the clamp, all subjects are counseled concerning the symptoms of hypoglycemia and are asked to report immediately any symptoms.  Subjects are observed on the CRC for at least 20 minutes following discontinuation of glucose infusion.

This procedure assumes that the hyperinsulinemic state is sufficient to completely suppress hepatic glucose production and that there is no net change in blood glucose concentrations under steady-state conditions. Under such conditions, the rate of glucose infused is equal to the rate of whole-body glucose disposal (GDR) or metabolizable glucose (M) and reflects the amount of exogenous glucose necessary to fully compensate for the hyperinsulinemia.

Indirect Calorimetry: A ventilated hood calorimeter (VMAX Encore; Carefusion Corp, Yorba Linda, CA) will be utilized to assess resting energy expenditure (REE) and respiratory quotient (RQ) by determining oxygen consumption (VO2) and carbon dioxide production (VCO2) for 20 minutes under fasting conditions before the clamp. REE will be normalized to lean body mass as determined by dual-energy x-ray absorptiometry.

Compensation: Recipients will be compensated $750 for completion of the study. $100 for completing the baseline visit, $400 for completing the week 6 visit ($100 for the clamp visit, $50 per capsule administration visit), and $250 for the week 12 visit. If subjects choose to participate in the optional extension study, they will be compensated an additional $100 ($25 for 6 month visit and $75 for 12 month,visit).They will also receive either a parking voucher or up to $20 to cover travel expenses at each visit

**D. Safety Monitoring**

Obese recipients will be monitored for clinical safety by history (and standard exams when seen in person) by asking open ended and then directed questions. Subjects will be followed closely, as noted above. Follow up calls made by a study coordinator will be documented and follow up information is reviewed on the same day with a study physician if any symptoms of concern are identified. A physician plan for ongoing follow-up will be noted and implemented. In any case of adverse events identified as grade 2 (moderate severity) an in-person visit with an investigator, either in the GI or ID outpatient practices, will be encouraged.

An AE will be considered related if, in the judgment of the treating physician it is both temporally related to administration of the capsules, and there is no other reasonable alternative explanation, and the AE is not more reasonably attributed to progression of the underlying illnesses.

Any serious adverse event (SAE) assessed as possibly, probably, or definitively related to fecal transplantation will be reported in an expedited fashion to the IRB and FDA. SAE’s are defined as any Grade 3 or 4 events as described on the attached AE grading chart, in addition to any event temporally associated with the study procedure that:

- results in death;
- is life threatening (places the subject at immediate risk of death from the event as it occurred);
- requires inpatient hospitalization or prolongation of existing hospitalization;
- results in a persistent or significant disability/incapacity;
- results in a congenital anomaly/birth defect;
- any other adverse event that, based upon appropriate medical judgment, may jeopardize the subject’s health and may require medical or surgical intervention to prevent one of the outcomes listed above.

Investigator monitoring with IRB and FDA oversight/reporting is proposed.

**E. Study Stop Rules**

The study will have a stop/safety pause in the event of any probably or definitely related SAE, or any apparent transmission of enteric, viral, or other infection from a donor to recipient. (Given the careful screening of donors, we believe this is highly unlikely). A safety pause will also be initiated in the event of a clinically significant complication of ingestion of a large number of capsules, for example medication bezoar (not observed to date in over 125 C. difficile patients treated, many with concurrent GI disease). We think this is very unlikely, given that we have demonstrated capsule dissolution occurs reasonably rapidly at neutral pH (i.e. once caps reach the small intestine.) We will track but not specifically stop the study if the following possible expected events occur:

- Transient fever after FMT (up to 102.5^◦^ F for 12 hours or less); this is occasionally noted, particularly in younger patients.

**F. Individual Subject Stop Rules**

By individual, people will not continue dosing if the following happens:

- If the individual is unable to swallow at least 10 capsules at a sitting.  Twenty capsules at week 0 and 10 capsules in subsequent weeks are the minimums considered a "full dose" for treatment and analysis purposes.
- If subject requests withdrawal.
- If subject vomits with clinically evident aspiration (by exam or xray; not observed to date)
- If subject experiences an SAE attributed by the study physician to the capsules

**G. Sample Sharing**

We may share coded samples containing no PHI from both donors and recipients with academic and commercial collaborators researching the microbiome, obesity, insulin sensitivity and probiotics/ecobiotics. Both groups will be notified of this sharing using standard language in the consent forms. We have no current plans or funding for these analyses but wish to keep this option open.

**VI. Biostatistical Analysis**

Change in insulin sensitivity is the primary outcome and will be compared between placebo and FMT groups over 12 weeks using longitudinal regression models. Secondary outcomes including change in insulin sensitivity, lipids, CRP, and DXA body composition will be similarly investigated. This is a pilot study to determine feasibility and safety, and to obtain data for power calculations for a larger powered study. After accounting for 15% dropout rate, and assuming a standard deviation of insulin sensitivity of 30-45% and a two-sided alpha of 0.05, this pilot study (n=12 per group) has 80% power to detect a 40-60% difference in insulin sensitivity between groups, and a 30-45% difference from baseline within each group. After accounting for 15% dropout rate, and assuming a standard deviation of weight loss of 5% and a two-sided alpha of 0.05, this pilot study (n=12 per group) has 80% power to detect a 6.6% difference in weight loss between groups, and a 5% difference in weight loss from baseline within each group.

Fecal samples from lean donors and obese recipients will be stored, as noted above. If funding allows in the future, these samples will undergo microbial analysis using 16S ribosomal DNA-based identification and deep sequencing techniques. Changes in the composition of the gut microbiota and metagenomic pathways will be correlated with clinical changes in metabolism.

Bone mineral density (BMD) of the spine, hip, and whole body will be assessed at baseline and at 12 months. This pilot study will provide preliminary data to expore the associations between BMD and gut microbiota prior to manipulation and after FMT.

1. **Risks and Discomforts**

Capsule administration: The risks of FMT include transmission of an infectious agent from an unrelated individual, or the potential for acquisition of medical illnesses not currently understood to be related to altered GI flora (for example inflammatory conditions, obesity, etc). We believe these risks are low. Other possible risks include nausea, vomiting, diarrhea, fever (all rare based upon experience to date). The risk of bezoar is very low as capsules dissolve promptly in non-acidic environments. We believe there is no risk to placebo capsules which contain gelatin, cocoa, and the same liquid vehicle as FMT caps (IV grade saline and USP glycerol).

Blood drawing: The amount of blood drawn for subjects completing the study is approximately 200 ml over a period of 12 weeks (or 260 mL over 12 months if subjects agree to enroll in optional substudy extension). This does not pose excessive risk to patients and are within Institutional Review Board guidelines. There is a small risk of bleeding, bruising and infection with phlebotomy.

Euglycemic Hyperinsulinemic clamp/insulin: Administration of insulin infusion can cause hypoglycemia. Blood sugar will be monitored every 5 minutes during the insulin clamp, and 20% dextrose is infused per protocol to achieve target blood glucose of 90mg/dL. In the event of hypoglycemia, 50% dextrose is available at the bedside, and a physician or nurse practitioner is present throughout the insulin clamp procedure. There is a minimal risk of burning when using a warming box. This is extremely rare. The warming box has an alarm that prevents the temperature from rising above the set limit.

DXA: Each whole body DXA scan delivers an effective radiation dose of 0.0084 mSv, and the hip and spine DXA scan delivers an additional 0.022mSv. The total amount of radiation is 0.0472 mSv over 12 weeks, or 0.0776 mSv over 12 months for those participating in the optional study extension.

Indirect Calorimetry: Indirect calorimetry for the measurement of resting energy expenditure is not known to cause any health risks.

**VIII. Potential Benefits**

It is hoped that subjects receiving FMT will experience weight loss and/or improvements in insulin sensitivity as a result of this intervention. Obese subjects may not benefit from being in the study. They can receive some medical information (labs, DXA) and will be referred to their physicians at the end of the study for continued care.

The study is likely to benefit the overall population of individuals with obesity, as it will contribute to an improved understanding of the physiologic and microbiomic mechanisms that control weight and glucose metabolism. An interesting scientific aspect of this study is analysis of the microbiome over time after prolonged FMT in obese but otherwise generally healthy subjects without a markedly altered microbiome at baseline. This may lead to future targeted therapies for weight loss and improving insulin sensitivity.

**IX. Monitoring and Quality Assurance**

Clinical data

The study coordinator and study physicians will carefully review testing on donors together, prior to sample collection, to ensure all inclusion criteria are met and that no exclusion criteria are met. This is also re-checked for capsules at the time of each administration with a Certificate of Analysis. Case report forms and follow-up questionnaires for recipients will be reviewed on a weekly or twice weekly basis for completeness and missing data points will be sought from records or phone contact if needed. Weight measurements and 48-hour food recalls will be evaluated by trained bionutritionists at the Clinical Research Center.

Capsule stability

We do not expect, and have not previously observed a decrement in the viability or efficacy of the capsules, which will be stored at -80 C in an alarmed freezer. Capsules will be discarded if not used by their expiration date. We have extensive experience with both frozen, and lyophilized bacterial vaccines for human use,[^30-3^](#_ENREF_29)^4^ and previously generated SOPs for short and long term analysis/stability testing of those mono-microbial frozen materials over time (under FDA IND # 12760 and 13937). Capsules which are not structurally intact cannot be used (see Certificate of Analysis). FMT Capsules are saved for analysis in the event of infection transmission. FMT Capsule lots where numbers allow have assessment of aerobic colony forming units over time (0 and 6 months) as a measure of stability. Placebo capsules containing frozen food grade material are not analyzed, and simply discarded at the expiration date.

**X. References**

1. Flint HJ. Obesity and the gut microbiota. *J Clin Gastroenterol.* Nov 2011;45 Suppl:S128-132.
2. Liou AP, Paziuk M, Luevano JM, Jr., Machineni S, Turnbaugh PJ, Kaplan LM. Conserved shifts in the gut microbiota due to gastric bypass reduce host weight and adiposity. *Sci Transl Med.* Mar 27 2013;5(178):178ra141.
3. Ridaura VK, Faith JJ, Rey FE, et al. Gut microbiota from twins discordant for obesity modulate metabolism in mice. *Science.* Sep 6 2013;341(6150):1241214.
4. Vrieze A, Van Nood E, Holleman F, et al. Transfer of intestinal microbiota from lean donors increases insulin sensitivity in individuals with metabolic syndrome. *Gastroenterology.* Oct 2012;143(4):913-916 e917.
5. Youngster I, Russell GH, Pindar C, Ziv-Baran T, Sauk J, Hohmann EL. Oral, capsulized, frozen fecal microbiota transplantation for relapsing Clostridium difficile infection. *JAMA.* Nov 5 2014;312(17):1772-1778.
6. Ogden CL, Carroll MD, Kit BK, Flegal KM. Prevalence of childhood and adult obesity in the United States, 2011-2012. *JAMA.* Feb 26 2014;311(8):806-814.
7. Beltran-Sanchez H, Harhay MO, Harhay MM, McElligott S. Prevalence and trends of metabolic syndrome in the adult U.S. population, 1999-2010. *J Am Coll Cardiol.* Aug 20 2013;62(8):697-703.
8. Lakka HM, Laaksonen DE, Lakka TA, et al. The metabolic syndrome and total and cardiovascular disease mortality in middle-aged men. *JAMA.* Dec 4 2002;288(21):2709-2716.
9. Festi D, Schiumerini R, Eusebi LH, Marasco G, Taddia M, Colecchia A. Gut microbiota and metabolic syndrome. *World journal of gastroenterology : WJG.* Nov 21 2014;20(43):16079-16094.
10. Backhed F, Ding H, Wang T, et al. The gut microbiota as an environmental factor that regulates fat storage. *Proceedings of the National Academy of Sciences of the United States of America.* Nov 2 2004;101(44):15718-15723.
11. Backhed F, Manchester JK, Semenkovich CF, Gordon JI. Mechanisms underlying the resistance to diet-induced obesity in germ-free mice. *Proceedings of the National Academy of Sciences of the United States of America.* Jan 16 2007;104(3):979-984.
12. Ley RE, Turnbaugh PJ, Klein S, Gordon JI. Microbial ecology: human gut microbes associated with obesity. *Nature.* Dec 21 2006;444(7122):1022-1023.
13. Armougom F, Henry M, Vialettes B, Raccah D, Raoult D. Monitoring bacterial community of human gut microbiota reveals an increase in Lactobacillus in obese patients and Methanogens in anorexic patients. *PLoS One.* 2009;4(9):e7125.
14. Santacruz A, Collado MC, Garcia-Valdes L, et al. Gut microbiota composition is associated with body weight, weight gain and biochemical parameters in pregnant women. *The British journal of nutrition.* Jul 2010;104(1):83-92.
15. Larsen N, Vogensen FK, van den Berg FW, et al. Gut microbiota in human adults with type 2 diabetes differs from non-diabetic adults. *PLoS One.* 2010;5(2):e9085.
16. Duncan SH, Lobley GE, Holtrop G, et al. Human colonic microbiota associated with diet, obesity and weight loss. *Int J Obes (Lond).* Nov 2008;32(11):1720-1724.
17. Schwiertz A, Taras D, Schafer K, et al. Microbiota and SCFA in lean and overweight healthy subjects. *Obesity (Silver Spring).* Jan 2010;18(1):190-195.
18. Turnbaugh PJ, Hamady M, Yatsunenko T, et al. A core gut microbiome in obese and lean twins. *Nature.* Jan 22 2009;457(7228):480-484.
19. Le Chatelier E, Nielsen T, Qin J, et al. Richness of human gut microbiome correlates with metabolic markers. *Nature.* Aug 29 2013;500(7464):541-546.
20. Cho I, Yamanishi S, Cox L, et al. Antibiotics in early life alter the murine colonic microbiome and adiposity. *Nature.* Aug 30 2012;488(7413):621-626.
21. Cox LM, Yamanishi S, Sohn J, et al. Altering the intestinal microbiota during a critical developmental window has lasting metabolic consequences. *Cell.* Aug 14 2014;158(4):705-721.
22. Trasande L, Blustein J, Liu M, Corwin E, Cox LM, Blaser MJ. Infant antibiotic exposures and early-life body mass. *Int J Obes (Lond).* Jan 2013;37(1):16-23.
23. Thuny F, Richet H, Casalta JP, Angelakis E, Habib G, Raoult D. Vancomycin treatment of infective endocarditis is linked with recently acquired obesity. *PLoS One.* 2010;5(2):e9074.
24. Vrieze A, Out C, Fuentes S, et al. Impact of oral vancomycin on gut microbiota, bile acid metabolism, and insulin sensitivity. *J Hepatol.* Apr 2014;60(4):824-831.
25. Turnbaugh PJ, Ley RE, Mahowald MA, Magrini V, Mardis ER, Gordon JI. An obesity-associated gut microbiome with increased capacity for energy harvest. *Nature.* Dec 21 2006;444(7122):1027-1031.
26. Sjögren K, Engdahl C, Henning P, Lerner UH, Tremaroli V, Lagerquist MK, Bäckhed F, Ohlsson C. The gut microbiota regulates bone mass in mice. Journal of bone and mineral research. 2012 Jun 1;27(6):1357-67.
27. Vimaleswaran KS, Cavadino A, Berry DJ, Jorde R, Dieffenbach AK, Lu C, Alves AC, Heerspink HJ, Tikkanen E, Eriksson J, Wong A. Association of vitamin D status with arterial blood pressure and hypertension risk: a mendelian randomisation study. The lancet Diabetes & endocrinology. 2014 Sep 30;2(9):719-29.
28. Charles, JF, Ermann, JE, Aliprantis, AO. The intestinal microbiome and skeletal fitness: Connecting bugs and bones. *Clinical Immunology*, Dec. 2014; 159: 163–169.
29. Integrative HMPRNC. The Integrative Human Microbiome Project: dynamic analysis of microbiome-host omics profiles during periods of human health and disease. *Cell host & microbe.* Sep 10 2014;16(3):276-289.
30. DeFronzo RA, Tobin JD, Andres R. Glucose clamp technique: a method for quantifying insulin secretion and resistance. *Am J Physiol.* Sep 1979;237(3):E214-223.
31. Johnston BC, Kanters S, Bandayrel K, et al. Comparison of weight loss among named diet programs in overweight and obese adults: a meta-analysis. *JAMA.* Sep 3 2014;312(9):923-933.
32. Johnson PV, Blair BM, Zeller S, Kotton CN, Hohmann EL. Attenuated Listeria monocytogenes vaccine vectors expressing influenza A nucleoprotein: preclinical evaluation and oral inoculation of volunteers. *Microbiol Immunol.* May 2011;55(5):304-317.
33. Kotton CN, Hohmann EL. Enteric pathogens as vaccine vectors for foreign antigen delivery. *Infect Immun.* Oct 2004;72(10):5535-5547.
34. Kotton CN, Lankowski AJ, Scott N, et al. Safety and immunogenicity of attenuated Salmonella enterica serovar Typhimurium delivering an HIV-1 Gag antigen via the Salmonella Type III secretion system. *Vaccine.* Sep 11 2006;24(37-39):6216-6224.
